# Giant nonlinear Hall effect in isospin symmetry broken bilayer graphene

**DOI:** 10.1038/s41467-026-75919-4

**Published:** 2026-07-20

**Authors:** Hao Chen, Qifeng Hu, Prasun Boyal, Kian Ping Loh

**Affiliations:** Department of Chemistry, National University of Singapore, Singapore, 117543 Singapore

**Keywords:** Phase transitions and critical phenomena, Electronic properties and devices, Electronic properties and materials

## Abstract

Spontaneous isospin ordering fundamentally reshapes quantum nonlinear transport, yet the nature of nonlinear responses in strongly correlated and symmetry-broken quantum states remain largely unexplored. In this work, we investigate a regime where symmetry breaking is not static but emerges dynamically as a tunable property of the electronic system, driven by strong correlations. Using high-quality, dual-gated Bernal bilayer graphene, we observe a giant nonlinear Hall conductivity (9.1 µm S V⁻¹) that manifests strongly in a field-driven isospin-polarized phase. In this correlated regime, the nonlinear conductivity scales exponentially with the linear conductivity, in stark contrast to the quadratic scaling expected in conventional nonlinear systems. Combined with quantum oscillation measurements and self-consistent theoretical modelling, our results indicate that this giant response is closely linked to an interaction-driven valley-polarized state. Our findings establish spontaneous isospin symmetry breaking as an effective route to giant nonlinear quantum transport, where interaction-enhanced skew scattering emerges as the dominant transport mechanism.

## Abstract (web summary)

Spontaneous isospin symmetry breaking can strongly influence quantum nonlinear transport. Here, the authors observe a giant nonlinear Hall conductivity in dual-gated Bernal bilayer graphene that manifests strongly in a field-driven isospin polarized phase.

## Introduction

Nonlinear electrical transport has emerged as a powerful probe of quantum geometry in solids, with the Berry curvature dipole providing a unified semiclassical framework for time-reversal-symmetric systems lacking inversion symmetry^1–4^. This paradigm successfully accounts for a broad class of nonlinear Hall responses, where weakly interacting band structures and crystal-imposed symmetry breaking govern transport. However, nonlinear transport in strongly correlated quantum states, where symmetry breaking emerges spontaneously rather than being structurally imposed, remains largely unexplored.

Dirac multilayer graphene offers an exceptional platform to address this question. In these systems, electrons possess an isospin degree of freedom associated with valley, spin, and layer quantum numbers. While isospin degeneracy is preserved in non-interacting regimes, electron–electron interactions under a perpendicular displacement field can spontaneously lift this degeneracy, giving rise to symmetry-broken metallic, half-metallic, and superconducting phases^5–10^. Unlike conventional symmetry breaking dictated by lattice structure, isospin ordering in graphene is interaction-driven and tunable by carrier density and electric field, providing an additional control knob for electronic transport.

In half-metallic and partially isospin-polarized systems, electronic transport becomes restricted to a smaller subset of spin–valley flavors. This reduction in available conduction channels removes the averaging effect typically present over degenerate states, making transport highly sensitive to the quantum geometry of the remaining bands. Within this regime, Berry curvature no longer behaves as a smoothly varying background field. Instead, it can become sharply localized near band edges and singular points. Consequently, even minor changes in carrier density can lead to disproportionally large shifts in the integrated Berry flux. This effect points toward a significant enhancement of nonlinear transport responses, potentially exceeding predictions based on semiclassical models.

Conventional semiclassical theories predict a quadratic scaling between the second-order nonlinear conductivity and the linear Drude conductivity, under the assumption of a single dominant relaxation time and weak correlations^3^. Such scaling may be violated in interaction-driven isospin-broken states—where symmetry breaking, quantum geometry, and electronic correlations are intrinsically intertwined. Beyond interaction-driven symmetry breaking, microscopic coupling between internal quantum degrees of freedom can also promote unconventional nonlinear behavior. For Dirac fermions, the coupling between chirality and pseudospin has been shown to induce non-analytic particle–hole asymmetry in tunneling geometries, leading to strongly nonlinear conductance characteristics^11^. While such effects highlight the sensitivity of nonlinear responses to internal quantum structure, they are largely confined to engineered tunneling configurations. Whether similarly unconventional behavior can emerge intrinsically in bulk transport through spontaneous symmetry breaking and strong correlations remains an open question.

Here, we demonstrate the emergence of a giant nonlinear Hall effect in Bernal-stacked bilayer graphene (BBG) by engineering its isospin symmetry. By applying a perpendicular displacement field, we lift the fourfold isospin flavor degeneracy to create a two-fold degenerate, valley-polarized/partially isospin-polarized (PIP_2_) state. Within this broken-symmetry phase, we observe a colossal nonlinear Hall conductivity, reaching a magnitude of 9.1 µm S V⁻¹. This enhanced response is accompanied by a clear breakdown of the conventional semiclassical scaling relation linking the second-order nonlinear conductivity to the linear Drude conductivity. Instead of the expected quadratic dependence, our measurements reveal a distinct exponential scaling. This stark deviation signifies a distinct nonlinear transport regime in which interaction-driven skew scattering no longer follows the conventional semiclassical scaling relation that is dominated by a single relaxation time parameter τ.

## Results

**Fig. 1:**
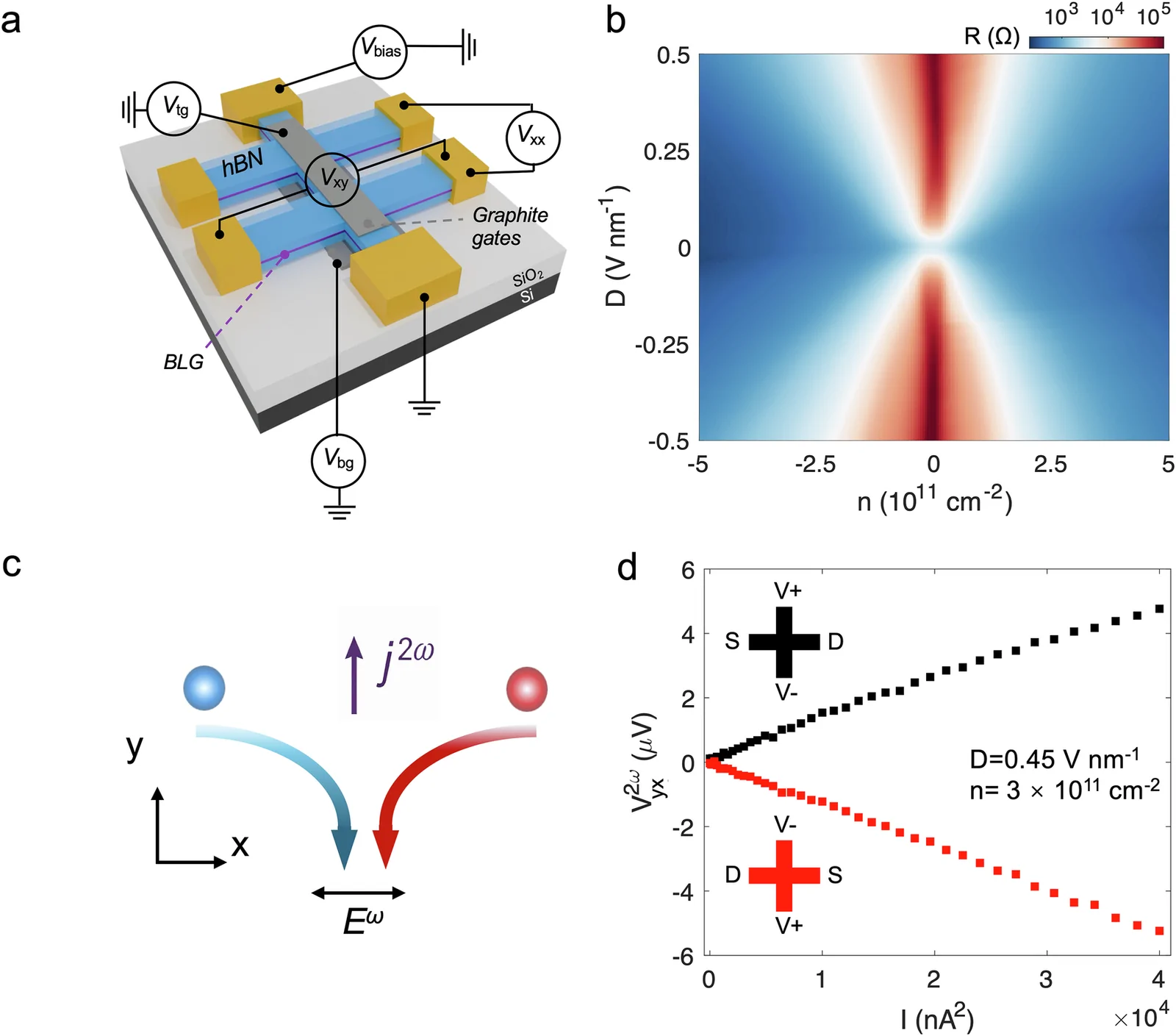
Device schematics and nonlinear Hall response in BBG. **a** Device schematic of a dual-graphite-gated bilayer graphene device in a Hall bar structure. Device components are indicated directly on layers or guided with gray dotted lines. Electrical measurement configurations are illustrated in black. **b** Carrier density and displacement field dependence of resistance (shown in log-scale) at 1.6 K. **c** Schematic illustration of the second-order Hall signal in the skew scattering regime. Blue and red trajectories denote electrons associated with opposite Berry curvature. **d** Second-order Hall signals at specific (*n*, *D*) conditions acquired in forward and backward setups, where the current direction and detection voltage probes are both reversed.

Observation of the broken isospin states often involves subtle energy scales that are easily masked by disorder or thermal effects and require highly sensitive detection methods at cryogenic temperature. Thus, a highly clean graphene system is needed for such a study above 1 K. The device structure is depicted in Fig. 1a. We fabricated a dual-gated Hall bar device utilizing hexagonal boron nitride (hBN) as the dielectric layer. Detailed information on the fabrication process and the electrical measurement setup can be found in the Methods. In Fig. 1b, we present a colormap of resistance (*R*_xx_) as a function of carrier density (*n*) and displacement field (*D*). The application of a finite displacement field results in the opening of a bandgap in BBG, illustrated by high-resistance states near the charge neutrality point (CNP). The electrical transport behavior of *R*_xx_ aligns with that expected for a Bernal-stacked bilayer graphene.

We did not observe any signature of half or full filling, which indicates the absence of moiré superlattices resulting from any unintentional alignment between the graphene and hBN flakes. Specifically, at *D* = 0.45 V nm^−1^ and *n* = 2 × 10^11^ cm^−2^, the second-order Hall ${V}_{{yx}}^{2\omega }$ signals exhibit a quadratic increase with the applied current, as showcased in Fig. 1d. To assess the reliability of the observed nonlinear Hall signals, we conducted measurements in both forward and backward current configurations, switching both the current direction and the detection probes. The black and red points in Fig. 1d correspond to the two measurement setups. The nonreciprocal relationship observed between the “forward” and “backward” conditions, together with the frequency independence (Supplementary Fig. 1), reinforces that the detected second-order Hall signals arise from a nonlinear origin, rather than from a diode effect.

To quantify the nonlinear response in BBG, we characterize its strength by ${\sigma }_{{yxx}}^{2\omega }=\frac{{J}_{y}^{2\omega }}{{{E}_{{xx}}^{\omega }}^{2}}=\frac{{V}_{{yx}}^{2\omega }}{{I}^{2}{R}^{3}}\frac{{L}^{3}}{{W}^{2}}$, where$\,{V}_{{yx}}^{2\omega },R$*, I, L,* and* W* denote second-order transverse voltage, resistance, current, length and width of the Hall bar device, respectively.

**Fig. 2:**
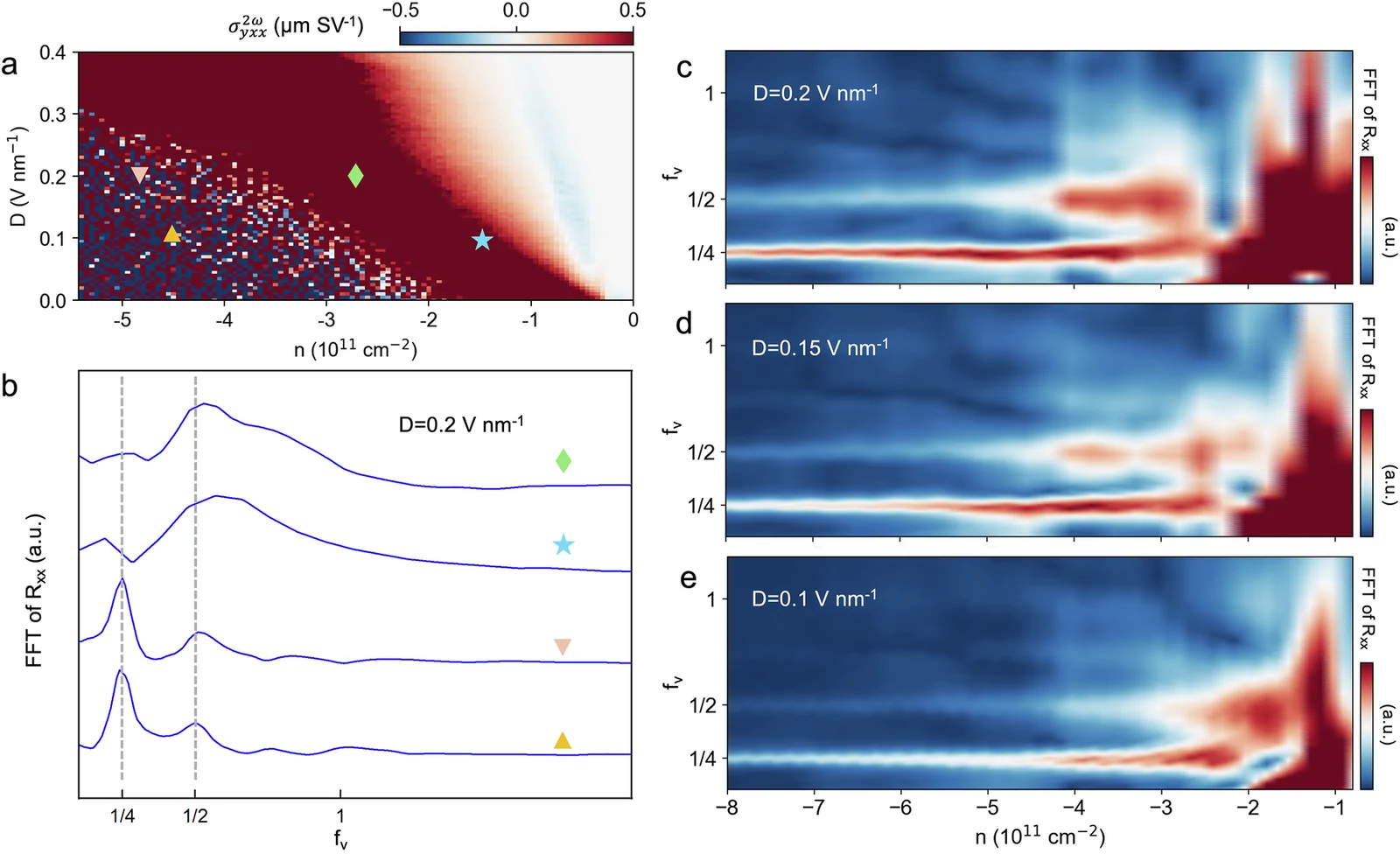
Nonlinear Hall signal in isospin polarized states. **a** Colormap of nonlinear Hall conductivity ${\sigma }_{{yxx}}^{2\omega }$ as function of *D*-field and carrier density n obtained at 1.6 K. **b** Fast Fourier transform (FFT) of quantum oscillations observed in specific (*n*, *D*) conditions denoted by the same symbols in (**a**). Pink triangle and green diamond denotes *n* = −4.8 × 10^11^ and −2.8 × 10^11^ cm^−2^ at *D* = 0.2 V nm^−1^, respectively. Yellow triangle and blue star denotes *n* = −4.5 × 10^11^ and −1.4 × 10^11^ cm^−2^ at *D* = 0.1 V nm^−1^, respectively. Filling fractions ¼ and ½ are marked with dashed lines. **c**–**e** FFT results colormap as a function of carrier density *n* at different *D*-field conditions, i.e., *D* = 0.2 V nm^−1^ (**c**), *D* = 0.15 V nm^−1^ (**d**), *D* = 0.1 V nm^−1^ (**e**), respectively.

We then obtain the dependence of ${\sigma }_{{yxx}}^{2\omega }$ on *D* and *n* as shown in Fig. 2a. Here we focus on the hole side and positive displacement field; the remaining parameter space is discussed in Supplementary Figs. 3 and 4. First, we observe that nonlinear Hall conductivity ${\sigma }_{{yxx}}^{2\omega }$ reaches 9.1 µm S V^−1^. This magnitude exceeds that of pristine WTe_2_^1,2^ and MoTe_2_^12^ crystals by several orders of magnitude and is on par with the Moiré-engineered graphene^13–17^ devices. A recent independent study has reported a comparable nonlinear Hall magnitude at the PIP2 state in BBG^18^, consistent with our observation.

At a fixed *D*-field, the nonlinear conductivity ${\sigma }_{y{xx}}^{2\omega }$ has negligible values at small carrier densities. ${\sigma }_{y{xx}}^{2\omega }$ slowly increases with carrier density and undergoes giant enhancement at intermediate carrier density. Finally, ${\sigma }_{y{xx}}^{2\omega }$ quenches to the noise level at elevated densities. The observed fluctuations in the high-density regime originate from the vanishingly small ${V}_{yx}^{2\omega }$ that approaches the detection limit of our instrument. This phenomenology, a gate-tuned emergence and suppression of a correlated state, is strongly reminiscent of broken-symmetry phases observed in multilayer graphene systems^5–7^.

To further investigate the nature of the electronic state in the critical region, we examine the period of quantum oscillations under an applied magnetic field. By performing Fourier analysis of the magnetoresistance *R*ₓₓ (1/B)^5,10^ (Supplementary Fig. 2), we extract the oscillation frequency, f_B_, which is directly proportional to the *k*-space size of the Fermi pocket f_v_ = f_B_/*n*φ_0_, where φ_0_ is the flux quantum. Here, f_v_ corresponds to the fraction of the total Fermi sea area enclosed by the Fermi surface, and 1/f_v_ indicates the energy degeneracy.

Fast Fourier transform (FFT) analysis of the quantum oscillations at selected (*n*, *D*) conditions in different areas in Fig. 2a is shown in Fig. 2b. The result reveals distinct isospin polarized states, i.e., partially isospin polarized state PIP_2_ (f_v_ = 1/2) and full metal state Sym_4_ (f_v_ = 1/4). Detailed FFT results of quantum oscillation at varying carrier density are obtained for each fixed *D* conditions (*D* = 0.1, 0.15, 0.2 V nm^−1^) and shown in Fig. 2c–e, respectively. For instance, it is observed that in Fig. 2c the f_v_ peak positions gradually shift from ½ to ¼ with increasing densities. The concurrent isospin degeneracy recovery and vanishing nonlinear responses agree well with a symmetric state. In the higher-density Sym_4_ regime, the measured second-harmonic Hall voltage becomes much weaker and approaches the instrument detection limit, so the extracted nonlinear conductivity is correspondingly noisier. For this reason, our data do not support a definitive analysis of the intrinsic nonlinear response in the Sym_4_ phase. We therefore focus on the PIP_2_ regime, where the giant nonlinear Hall signal is clear and reliably resolved in our device. At the isospin transition from degenerate full metal to non-degenerate half-metal state at *D* = 0.2 V nm^−1^, there is a giant increase in nonlinear second-order conductivity ${\sigma }_{{yxx}}^{2\omega }$ by around three orders of magnitude. The nonlinear Hall response exhibits similar behavior at negative *D*-field and hole side (Supplementary Fig. 3), where the strongest enhancement likewise appears in the PIP2 regime at intermediate carrier densities.

It is observed that the nonlinear response at the electron side (Supplementary Fig. 4) is an order of magnitude smaller than that in the hole side. This weak response is accompanied by the absence of a clear PIP_2_ signature in the corresponding FFT analysis (Supplementary Fig. 4b, c). Taken together, these observations show that the giant nonlinear Hall response in our device is most prominently associated with the emergence of the PIP_2_ state, while also highlighting the pronounced electron–hole asymmetry of Bernal bilayer graphene.

**Fig. 3:**
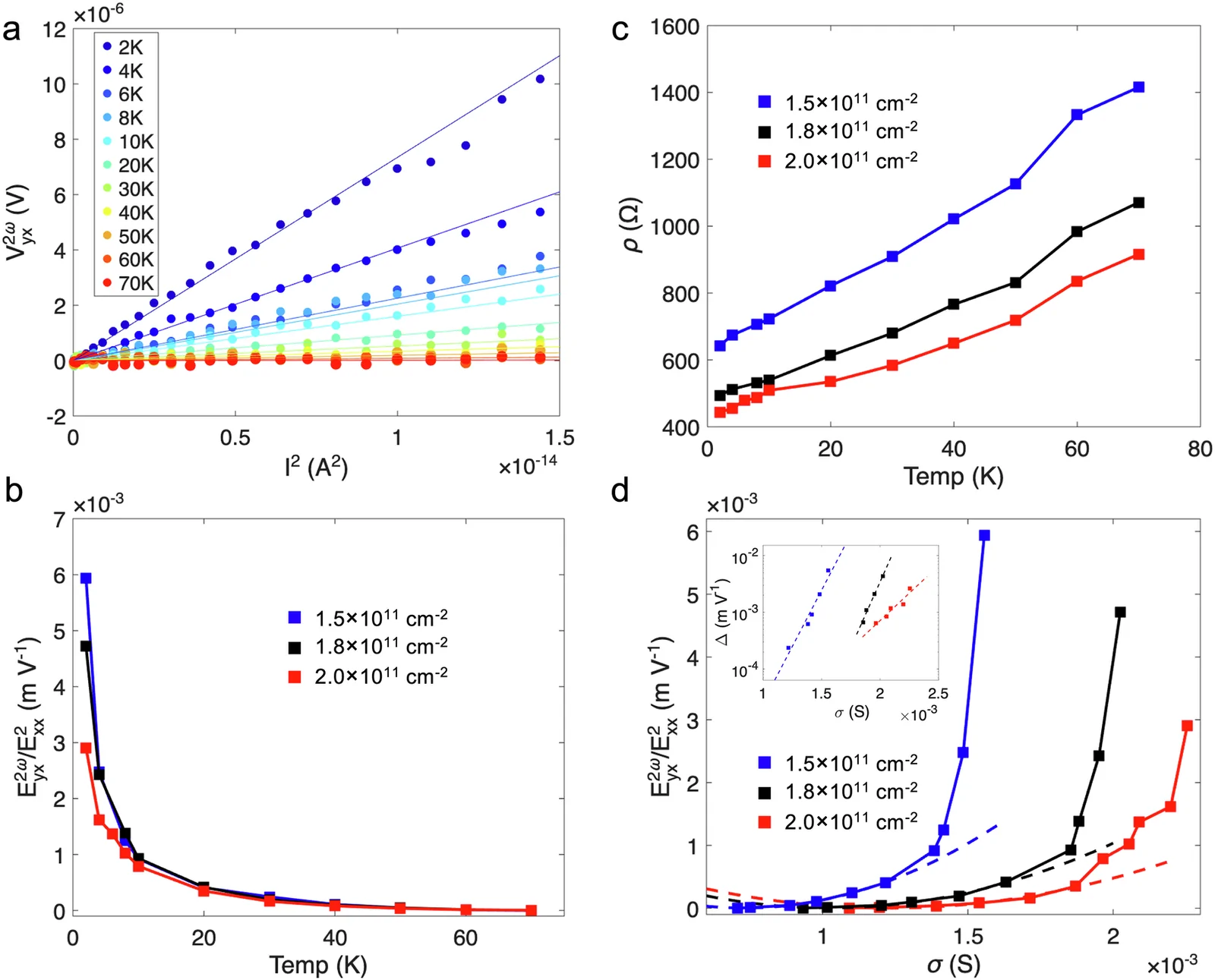
Temperature dependence of the nonlinear Hall effect. **a** Temperature dependence of ${V}_{{yx}}^{2\omega }$ at different carrier densities at *n* = 1.8 × 10^11 ^cm^−2^. Solid lines are linear fits to each data set at fixed temperatures. **b**, **c** ${E}_{{yx}}^{2\omega }/{E}_{{xx}}^{2}$ and resistivity as function of temperature for different carrier densities *n* = 1.5, 1.8 and 2.0 × 10^11^ cm^−2^, respectively. **d** Scaling relation between ${E}_{{yx}}^{2\omega }/{E}_{{xx}}^{2}$ and conductivity at corresponding carrier densities. The fitted parabolic curves (dashed lines) are obtained using data points at temperatures larger than 10 K. All results are acquired at *D* = 0.45 V nm^−1^. Inset: Extracted Δ as a function of conductivity, plotted in log scale.

To elucidate the microscopic origin of the observed nonlinear response, we analyzed the scaling relationship between the second-order nonlinear conductivity and the linear conductivity through temperature-dependent measurements. In Fig. 3a, the second-harmonic Hall voltage ${V}_{{xy}}^{2\omega }$ is plotted against the square of the current at various temperatures within the partially isospin-polarized (PIP2) states. A linear dependence is observed across all temperatures, with the slopes decreasing as temperature increases.

The strength of the second-order nonlinear response can be quantified using the ratio of electric fields, expressed as:$\frac{{\sigma }_{{yxx}}^{2\omega }}{\sigma }=\frac{{E}_{{yx}}^{2\omega }}{{E}_{{xx}}^{2}}=\frac{{V}_{{yx}}^{2\omega }{L}^{2}}{{V}_{{xx}}^{2}W}$ Using this relation, we extracted the field ratios ${E}_{{yx}}^{2\omega }/{E}_{{xx}}^{2}$ under fixed carrier density n and displacement field D conditions, as shown in Fig. 3b. This ratio decreases monotonically with rising temperature. Complementary to these results, the temperature dependence of the resistivity is presented in Fig. 3c.

To clarify the microscopic origin of the nonlinear signals, it is essential to examine the scaling relationship with the linear conductivity, σ, as established in prior theoretical work^3^. To this end, we plot the ratio ${E}_{{yx}}^{2\omega }/{E}_{{xx}}^{2}$ against σ in Fig. 3d. According to theoretical models³^,^¹⁹, the semiclassical scaling relationship between ${E}_{{yx}}^{2\omega }/{E}_{{xx}}^{2}$ and conductivity can be expressed as:

$\frac{{E}_{{yx}}^{2\omega }}{{E}_{{xx}}^{2}}={A}_{2}{\left(\frac{\sigma }{{\sigma }_{0}}\right)}^{2}+{A}_{1}{\left(\frac{\sigma }{{\sigma }_{0}}\right)}^{1}+{A}_{0}$ where ${A}_{i}$ (for $i={\mathrm{0,1,2}}$) are scaling coefficients, σ is the conductivity, and ${\sigma }_{0}$ represents the conductivity in the zero-temperature limit.

In contrast to previous experimental observations that showed a quadratic scaling between ${E}_{{yx}}^{2\omega }/{E}_{{xx}}^{2}$ and conductivity, the relationship observed here does not conform to this conventional behavior. Specifically, the scaling follows a parabolic trend in the low-conductivity (high-temperature, >10 K) region, but deviates significantly at high conductivity (low-temperature, <10 K).

To isolate this deviation, we subtract the parabolic contribution from the nonlinear strength in the high-conductivity regime using: $\Delta=\frac{{E}_{{yx}}^{2\omega }}{{E}_{{xx}}^{2}}-\left(A{\sigma }^{2}+B\sigma+C\right).$ As shown in the inset of Fig. 3d, the extracted Δ exhibits an approximately linear dependence on a log scale, indicating that the low-temperature nonlinear response acquires an exponential dependence on conductivity. This unconventional scaling behavior^19^ suggests that the nonlinear transport here is not governed by a single parameter, such as the relaxation time, as assumed by the classical Drude model^3,20^. We interpret this anomaly primarily in terms of correlation-enhanced skew scattering associated with the valley-polarized state. At higher temperatures, the data align more closely with the expected parabolic trend, reflecting a crossover from a strongly enhanced low-temperature response to a weaker residual nonlinear background. In the displacement-field-driven isospin symmetry-breaking regime, Berry curvature becomes highly nonuniform. Tuning the carrier density into the partially isospin-polarized (PIP2) state positions the Fermi level within regions of intense Berry curvature accumulation, without cancellation from the opposite valley. Even small shifts in carrier density can dramatically alter the net Berry flux, leading to substantial enhancements in nonlinear conductivity.

This anomalous scaling behavior is consistently observed across multiple devices and under complementary tuning protocols: varying carrier density at fixed displacement field (Supplementary Fig. 5b) and varying displacement field at fixed carrier density (Supplementary Fig. 5a). The reproducibility of these results confirms that the unconventional scaling is not an artifact of specific parameters or device variability, but a robust signature of nonlinear transport in the PIP2 regime.

We further note that the nonlinear response in two dimensions is described by a rank-3 tensor. In another device, both transverse and longitudinal second-order responses are observed (Supplementary Fig. 7). The longitudinal response is finite and broadly distributed in the phase space, whereas the transverse nonlinear Hall response is more localized and phase-selective. However, the Hall-bar geometry accesses only limited combinations of the rank-3 nonlinear conductivity tensor and does not allow a full tensor reconstruction. In addition, the longitudinal second-harmonic signal is more susceptible to parasitic thermoelectric contributions, whereas the transverse channel provides a cleaner and more phase-sensitive probe of the symmetry-broken nonlinear response. We therefore focus our analysis on ${V}_{{yx}}^{2\omega }$.

**Fig. 4:**
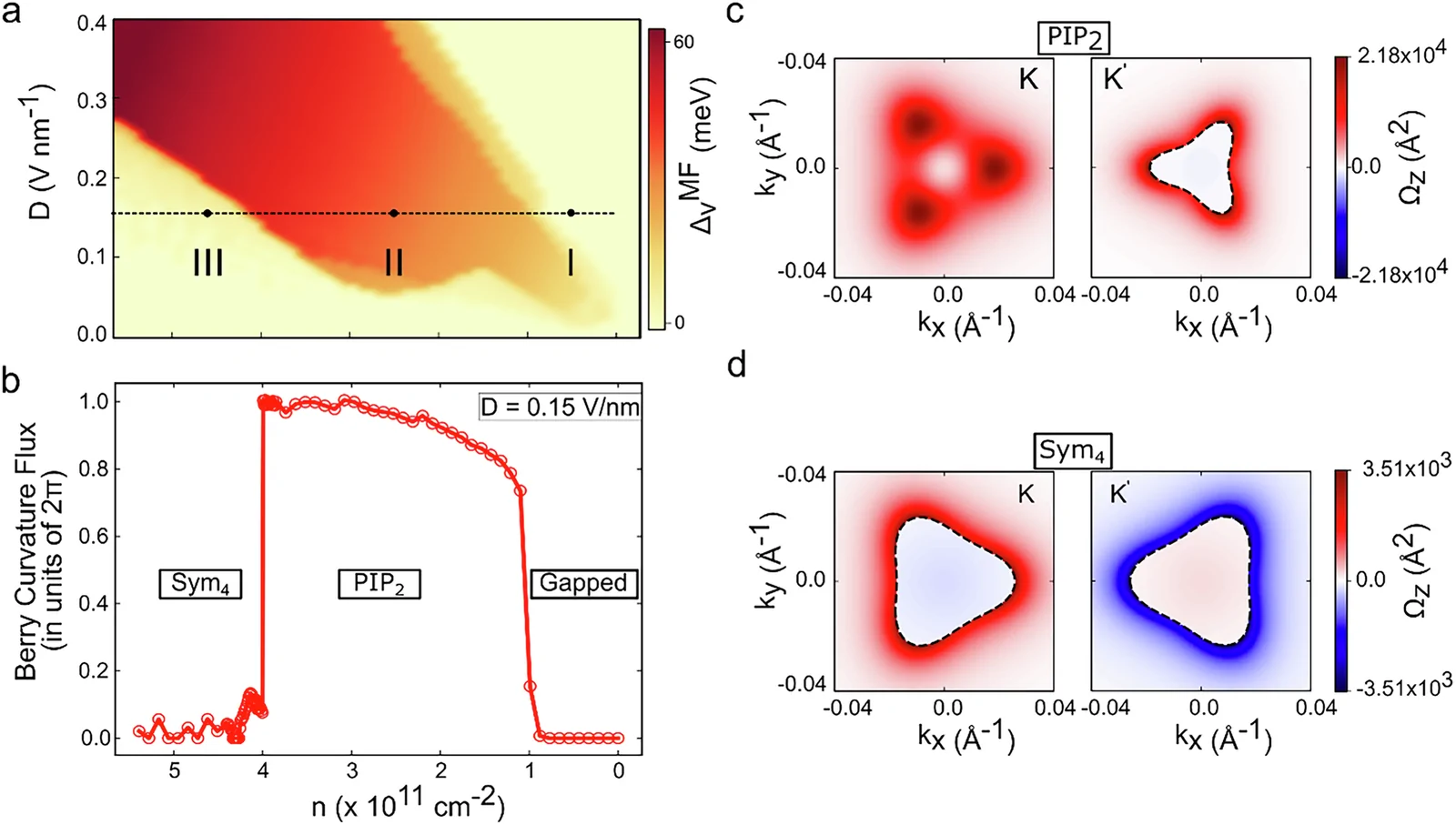
Valley polarization, Berry curvature flux, and their connection to the nonlinear Hall effect. **a** Valley polarization gap ${\Delta }_{v}$ as a function of carrier densityn and displacement field D, showing three distinct regions. **b** Total Berry curvature flux integrated over occupied states as a function of n at fixed *D* = 0.15 V nm^−1^, with three different regions: a gapped phase with vanishing flux, a half-metallic (PIP_2_) phase where the flux approaches 2π, and a symmetric metallic (Sym4) phase. **c**, **d** Momentum-resolved Berry curvature with Fermi surface shown as black dashed line in the PIP_2_ and Sym_4_ state, respectively.

To elucidate the observed anomalous nonlinear transport behavior, we conducted energy band calculations that incorporate mean-field interaction terms. The resulting electronic phase evolution as a function of carrier density n and displacement field D is summarized in Fig. 4a, where the valley polarization gap ${\Delta }_{v}$ delineates three distinct regions that align closely with the experimental nonlinear Hall (NLH) signals. In Region I, corresponding to very low carrier densities, the system remains fully gapped with no discernible Fermi surface, indicating an insulating phase where conventional electronic transport is suppressed. As the carrier density increases, the system enters the interaction-driven PIP₂ phase. In this regime, the intervalley exchange interaction ${U}_{{\mbox{valley}}}$ induces spontaneous valley symmetry breaking: one valley becomes fully occupied while the other remains partially filled, lifting the time-reversal symmetry (TRS) of the system. Despite this broken TRS in the electronic structure, we observe no discernible zero-field linear anomalous Hall signal within our experimental sensitivity (Supplementary Fig. 9). This suggests that the PIP₂ phase manifests more prominently through the nonlinear Hall channel than through the linear Hall response. At even higher densities, the system transitions into the symmetric full-metal (Sym4) phase, characterized by a vanishing valley polarization gap and the restoration of balanced occupation between the K and K′ valleys.

We find that the calculated mean-field valley splitting ${\Delta }_{v}^{{MF}}$ is on the order of tens of meV, which is significantly larger than the estimated valley gap of $\sim {k}_{B}T$ (~0.69 meV) obtained from temperature-dependent NLH measurements (Fig. 3d and Supplementary Fig. 5). Since NLH measurements probe only low-energy carriers near the Fermi surface, this discrepancy suggests a substantial reduction in the effective valley gap $({\Delta }_{v}^{{\mbox{eff}}})$ This reduction may arise from several factors, including dynamical screening by mobile carriers, thermal smearing, and quantum fluctuations not captured by the mean-field approximation. These effects can suppress the valley imbalance for low-energy electrons, as discussed in refs. ^21–23^.

Within the skew-scattering framework, the transport response across these phases is fundamentally tied to the evolution of the total Berry curvature flux(Φ). As illustrated in Fig. 4b, Φ is plotted as a function of n at a fixed D = 0.15 V nm^−1^. In the initial gapped phase, Φ remains strictly zero, consistent with the TRS constraints of gapped bilayer graphene. When transitioning into the PIP_2_ region, Φ increases rapidly to a quantized value of 2π. Remarkably, the transition into the Sym_4_ phase is characterized by an abrupt drop in Φ, indicating a sharp transition in the Berry curvature topology associated with the restoration of valley degeneracy. As shown in Fig. 4c, d, this behavior can be understood from the alignment of the Berry curvature in both valleys. In the PIP_2_ phase, the K valley is fully occupied while the K’ valley hosts a Fermi surface; the Berry curvature near this Fermi contour possesses the same sign as the K valley due to the broken TRS, leading to constructive addition. Conversely, in the Sym_4_ phase, both valleys possess Fermi surfaces with opposite Berry curvature signs, resulting in a significant reduction of the total flux.

Within this picture, the relationship between skew-scattering susceptibility and valley population imbalance arises from the asymmetric impurity scattering probabilities inherent to the valley’s internal quantum numbers. In a system with ${\Delta }_{{valley}}=0$, these asymmetric contributions cancel exactly; however, a finite population imbalance between the valleys allows the asymmetric scattering to generate a net transverse second-order current. However, this imbalance is thermally regulated and in the low temperature limit $({k}_{B}T\ll {\Delta }_{{valley}}),$ thermally activated minority valley population scales with the Boltzmann weight as ${n}_{{K}^{\prime}} \sim {e}^{-\frac{{\Delta }_{{\rm{valley}}}}{{k}_{B}{T}}}$ reflecting the exponential suppression of the higher-energy valley’s occupation. The net skew scattering rate is then expected to be inversely proportional to the minority valley contribution. Consequently, the skew-scattering contribution to the nonlinear Hall response in the TRS broken PIP_2_ state can be described by the modified scaling relation:${\sigma }_{{skew}}^{2\omega } \sim {\sigma }_{{xx}}^{2}\exp ({\Delta }_{{valley}}/{k}_{B}T){{\boldsymbol{.}}}$ As the longitudinal conductivity ${\sigma }_{{xx}}$ does not depend on the valley population imbalance and its temperature dependence only comes from the carrier mobility for fixed carrier densities, the exponential factor can become the dominant factor at low temperature leading to the observed exponential enhancement of NLH signal, as shown in Fig. 3d. Whereas in the full metallic Sym_4_ phase, the absence of valley imbalance due to restoration of the valley degeneracy is expected to strongly suppress this exponential contribution. We emphasize that this skew-scattering description is a physically motivated interpretation rather than a unique microscopic mechanism. Recent theoretical work^24^ has also shown that nonlinear transport in symmetry-broken systems can contain disorder-related side-jump- and skew-scattering-type contributions at the same order as quantum-geometric dipole terms, highlighting the need to go beyond a purely intrinsic dipole description.

The Berry curvature dipole model, while successful, is fundamentally limited by its reliance on weak electron correlations, non-interacting band structures, and extrinsic, crystal-built-in symmetry breaking. Our work extends this paradigm to a regime where symmetry breaking is not fixed but instead emerges as a dynamic and tunable property arising from strong electron correlations.

In this work, we have observed a giant nonlinear Hall response in Bernal bilayer graphene that arises without the need for moiré engineering or mechanical strain^14,17,25,26^. This response is enabled by spontaneous isospin symmetry breaking and can be precisely tuned via carrier density and displacement field. The second-order nonlinear Hall conductivity reaches 9.1 µmSV^−^¹ in BBG—orders of magnitude larger than that of pristine inversion-broken crystals such as WTe₂ and MoTe₂, and comparable to values reported in engineered moiré systems. Furthermore, the scaling evolves from a conventional parabolic dependence to an anomalous exponential regime within the isospin-polarized half-metal (PIP_2_) state. In this correlated environment, the Drude conductivity scaling breaks down, and the interaction-enhanced skew scattering emerges as the dominant transport mechanism underlying the observed nonlinear Hall response. Our work establishes spontaneous isospin flavor symmetry breaking as a fundamental and broadly applicable mechanism for generating giant nonlinear quantum transport, opening pathways to explore nonlinear responses in strongly correlated Dirac systems.

## Methods

### Device fabrication

Bilayer graphene, graphite, and hBN flakes are exfoliated on SiO_2_/Si wafers. The whole heterostructure is assembled using a dry-transfer technique using a PC (polycarbonate) film/PDMS stamp. We first pick up the top graphite and subsequently top hBN, bilayer graphene, bottom hBN, and bottom graphite, and put the whole stack on another wafer. The hBN layers are intentionally misaligned with the bilayer graphene flake to avoid a moiré heterostructure. Graphite flakes of more than six layers are used as local gates to reduce disorder and ensure high-quality devices. Finally, the device was etched into a Hall bar and contacted using edge-contact with Cr (5 nm)/Au (70 nm).

### Transport measurements

Transport measurements were conducted in a variable-temperature insert in the Oxford Teslatron system. The standard lock-in technique is used to measure the four-probe voltages using SRS830. Low-pass filters are used to suppress low-frequency noises. A constant current source is applied with a range from 10 to 200 nA. Gate voltages are applied using Keithley 2400 and 6430.

### Theoretical calculations

The electronic structure of BBG was modeled using a low-energy Slonczewski–Weiss–McClure (SWMcC) Hamiltonian, incorporatingk.p hopping parameters $({\gamma }_{0},{\gamma }_{1},{\gamma }_{3},{\gamma }_{4})$ and a layer-asymmetric potential $\Delta={eDd} / \epsilon$ to account for the external displacement field D with d being the interlayer distance (see Supplementary Section VII). To capture electron–electron correlations, we supplemented the single-particle terms with mean-field Hubbard interactions for spins $({U}_{{spin}})$ and valleys $({U}_{{valley}})$. The interaction strengths were determined via first principles constrained Random Phase Approximation (cRPA) on a hBN encapsulated BBG, which yielded ${U}_{{valley}}=\,1.02\,{\rm{{eV}}}$ and ${U}_{{spin}}=0.53\,{\rm{{eV}}}$ at zero displacement field. The total Hamiltonian was solved self-consistently on a 300×300 *k*-point grid centered at the K and K’ valleys. Starting from initial polarization guesses, the spin and valley order parameters were iteratively converged within a tolerance of $0.1\,{\rm{{meV}}}$. The resulting converged eigenstates were then used to calculate the Berry curvature flux and the Berry curvature via velocity operators across the $\left(n,D\right)$ parameter space.

## Supplementary information

Supplementary Information

Peer Review file

## Source data

Source Data

## Data Availability

The authors declare that the data supporting the findings of this study are available within the paper and its Supplementary Information files. Source data are provided with this paper.
