# Supplementary material for: Giant nonlinear Hall effect in isospin symmetry broken bilayer graphene
Source: Nat Commun. 2026 Jul 20;17:8856. doi: 10.1038/s41467-026-75919-4 (PMC13500830; doi:10.1038/s41467-026-75919-4)
Supplement: Supplementary file 1 [file 41467_2026_75919_MOESM1_ESM.pdf]

Supplementary Information for
Giant Nonlinear Hall effect in Isospin Symmetry Broken Bilayer Graphene

Hao Chen[1,†], Qifeng Hu[1,†], Prasun Boyal[1,†], and Kian Ping Loh[1,*]

[1] Department of Chemistry, National University of Singapore, Singapore, 117543, Singapore.

† These authors contribute equally to the work.
* chmlohkp@nus.edu.sg

Supplementary Section I: Frequency independence of NLH signal

To validate the origin of the NLH signal, AC frequency dependence of measured nonlinear voltage signals is presented in Supplementary Figure 1. Consistent signals are independent of signal frequencies, excluding diode effect.

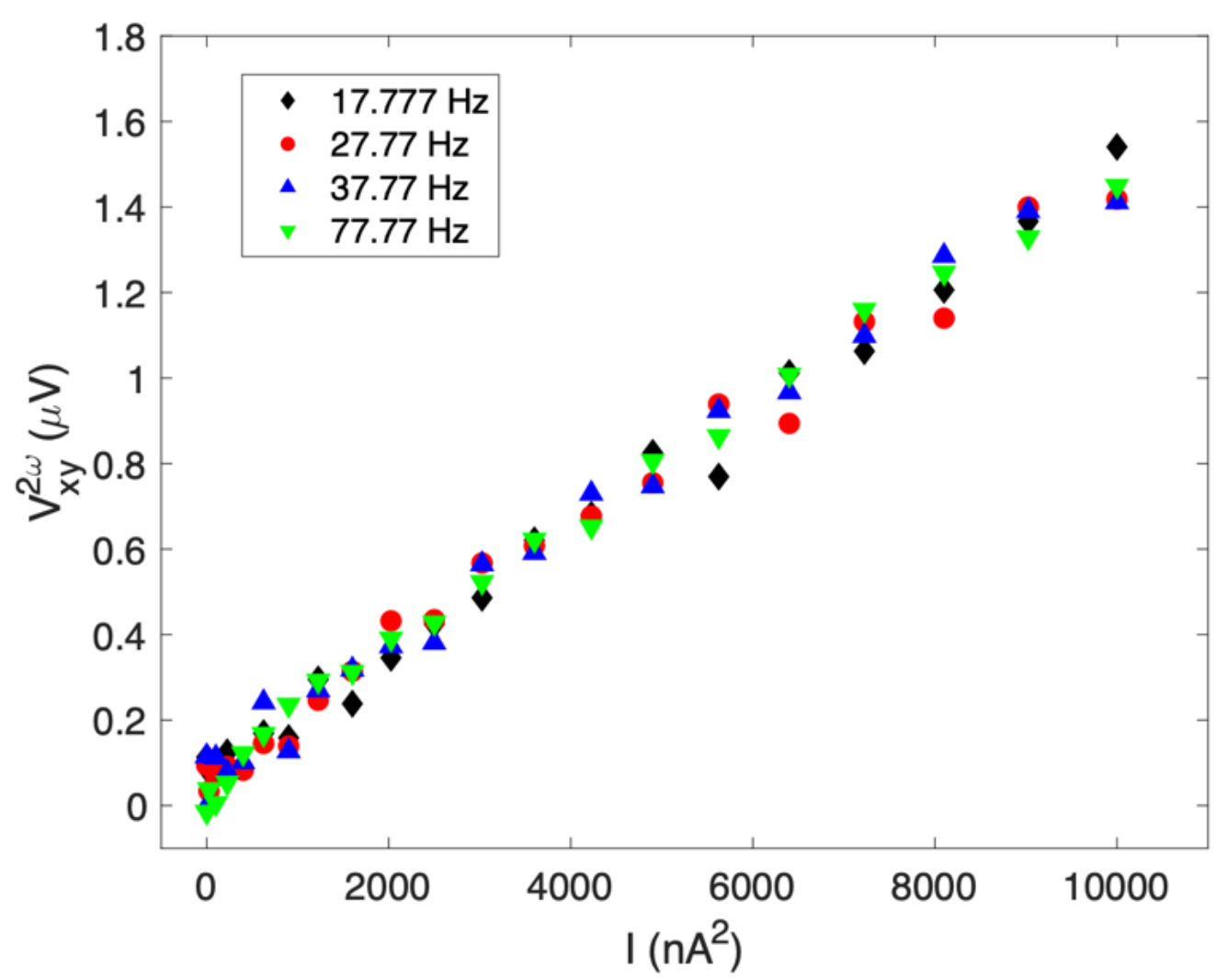

Supplementary Figure 1. Frequency dependence of nonlinear Hall voltages in BBG. Insets denote different frequencies at which second order voltages signals are obtained at a specific (n, D) condition.

Supplementary Section II: Quantum oscillations and FFT analysis

FFT analysis of Shubnikov de Haas oscillations are obtained in the following approach. First, the magnetoresistance data are obtained at fixed (n, D) conditions denoted in Supplementary Fig. 2a&c. Then the background is subtracted from the R_{xx}(1/B) data following the method described in Ref. 1. The data is then interpolated to produce an even grid as a function of 1/B (Supplementary Fig. 2b&d). Then the extracted frequency (f_B) and magnitude from FFT is factorized through $f = f_B/n\varphi_0$ and shown in Figure 2 in the main text.

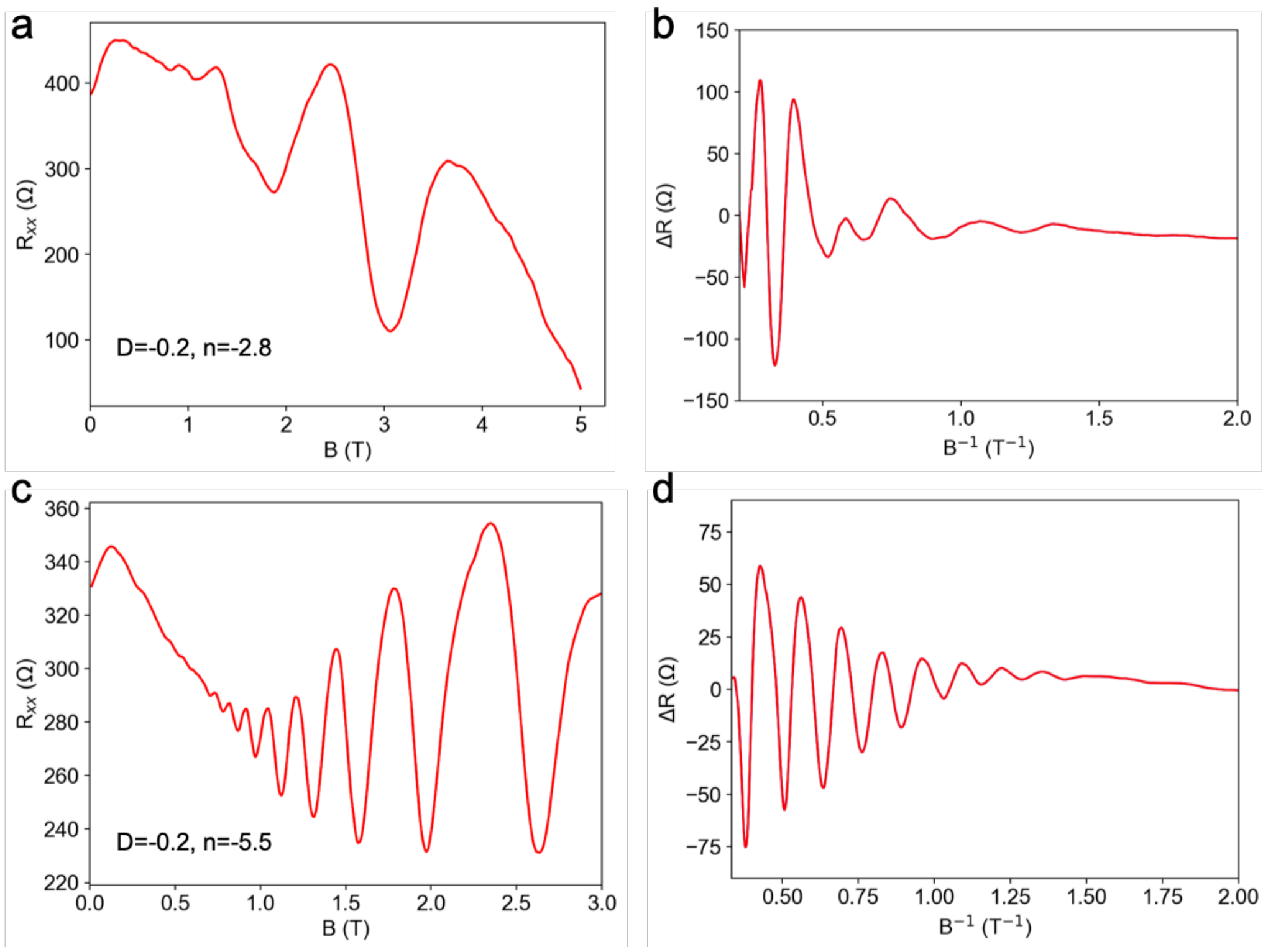

Supplementary Figure 2. Quantum oscillations at different (*n*, *D*) conditions. Quantum oscillations at two distinct isospin symmetric conditions are shown in **a** and **c,** respectively. Extracted variation of resistance of **a** and **c** after background removal are shown in **b** and **d**, respectively.

Supplementary Section III: Nonlinear response in the -D field hole side.

In addition to Figure 2, the NLH $\sigma_{yxx}^{2\omega}$ in the negative displacement field region and corresponding FFT results are presented in analyzed in Supplementary Figure 3.

As shown in Supplementary Figure 3a, for a fixed D-field, $\sigma_{yxx}^{2\omega}$ first takes tiny value at small carrier density, then grows to giant values at intermediate density and finally enters noisy region with fluctuating amplitude as $V_{yx}^{2\omega}$ signal approaches instrument resolution while finite V_{xx} amplifies the noise signal. This transition is also on par with the behaviour of $\sigma_{yxx}^{2\omega}$ in the +*D*-field region in Figure 2a. In Supplementary Figure 3b,c&d, FFT results of R_{xx} in such conditions also illustrate the transition from half metal to full metal state. The half metal regions are generally consistent with the finite $\sigma_{yxx}^{2\omega}$ region in Supplementary Figure 3a. The inset dashed line highlights the shift of half metal region with increasing |*D*|.

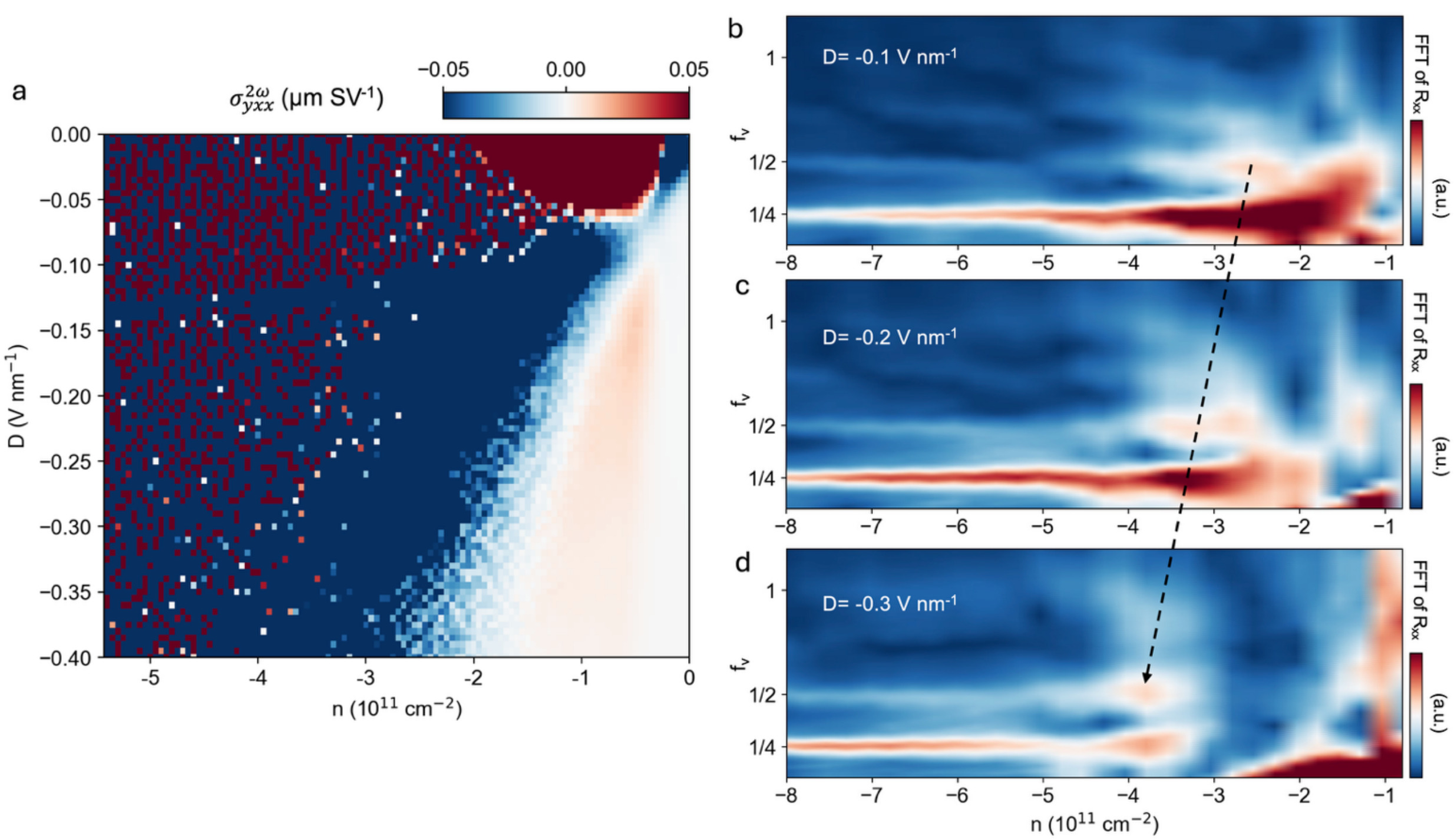

Supplementary Figure 3. Nonlinear response and FFT analysis in the -D field and hole side. a, Colormap of nonlinear Hall conductivity $\sigma_{yxx}^{2\omega}$ as function of D-field (negative region) and carrier density n obtained at 1.6 K. **b,c&d**, FFT results colormap as function of carrier density n at different D-field conditions, i.e., *D*= -0.1 V nm^{-1} (**b**), *D*= -0.2 V nm^{-1} (**c**) and *D*= -0.3 V nm^{-1} (**d**) , respectively. The dashed line is a guide to the eye to the shift of the f_v=1/2 peaks with increasing *D*-field magnitude.

Supplementary Section IV: Nonlinear response in the +D field electron side.

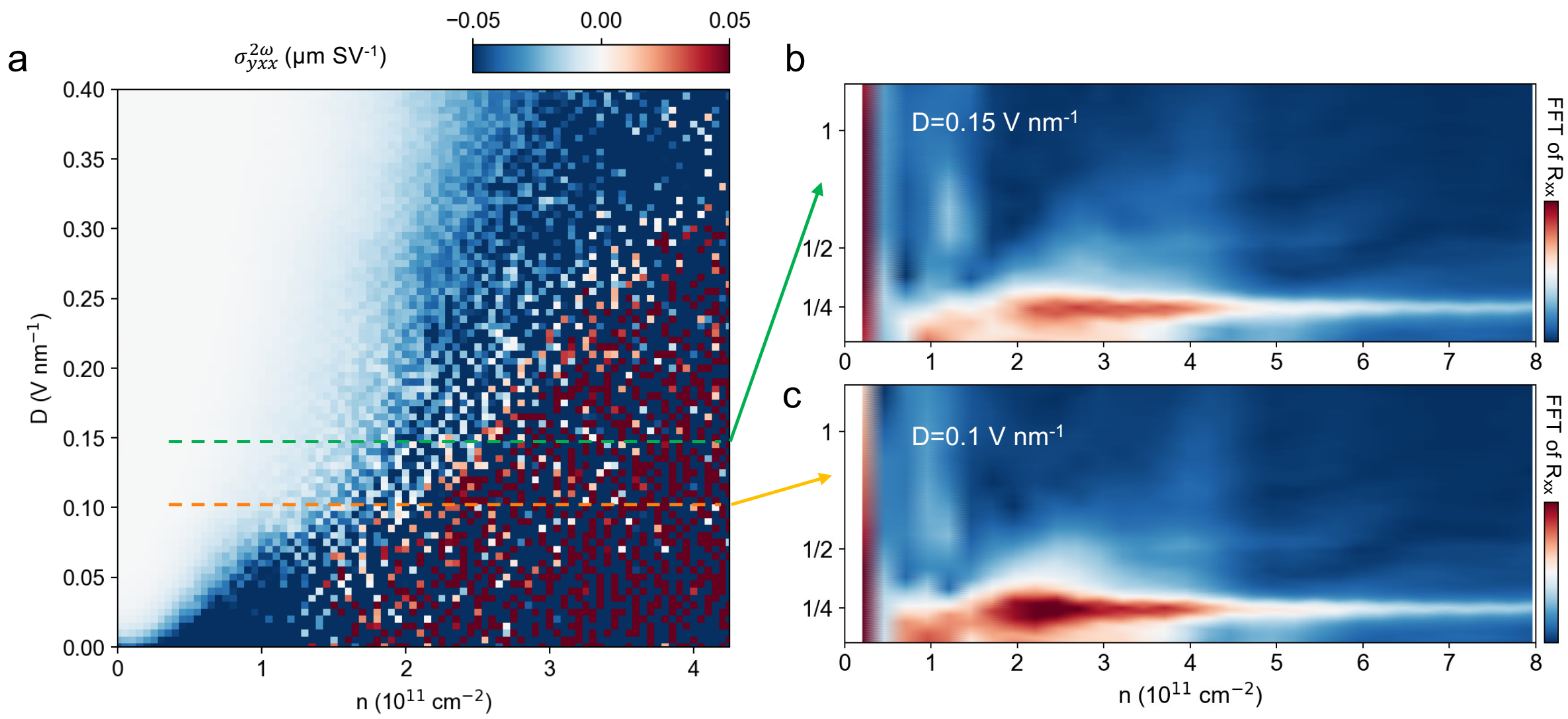

Supplementary Figure 4. Nonlinear response and FFT analysis in the +D field electron side. **a**, Colormap of nonlinear Hall conductivity $\sigma^{2\omega}_{yxx}$ as function of +*D*-field and carrier density n at electron side obtained at 1.6 K. **b&c**, FFT results colormap as function of carrier density n at different D-field conditions, i.e., *D*= 0.1 V nm^{-1} (**b**) and *D*= 0.15 V nm^{-1} (**c**) respectively. Apart from Sym_4 state (f_v=1/4), no signatures of f_v=1/2 peaks can be observed, i.e., isospin symmetry is preserved.

Supplementary Section V: Scaling relation in PIP2 state in additional devices.

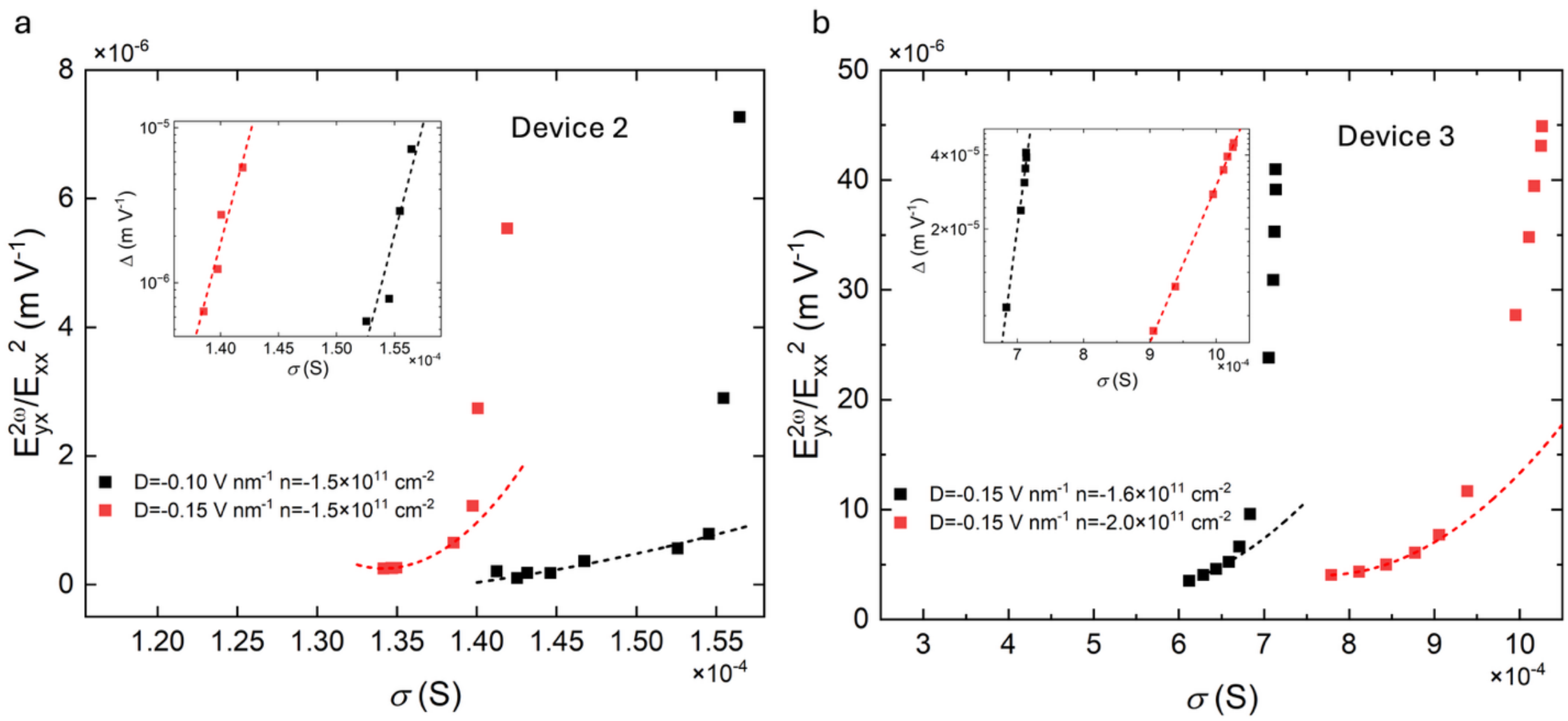

Supplementary Figure 5. Scaling anomaly in PIP2 state in additional devices. Nonlinear Hall strength as function of conductivity in device 2 (a) and device 3 (b), respectively. Dashed lines are parabolic fitting curves to the lower conductivity region (higher temperature range). Inset: log-scale plot of extracted signals as function of conductivity at higher conductivity (lower temperature range). The dashed line indicates linear dependence in such plot. Naively we can estimate the energy gap between such PIP2 state and full metal state to be around k_BT~0.69 meV.

Supplementary Section VI: Temperature dependence of NLHE in BBG

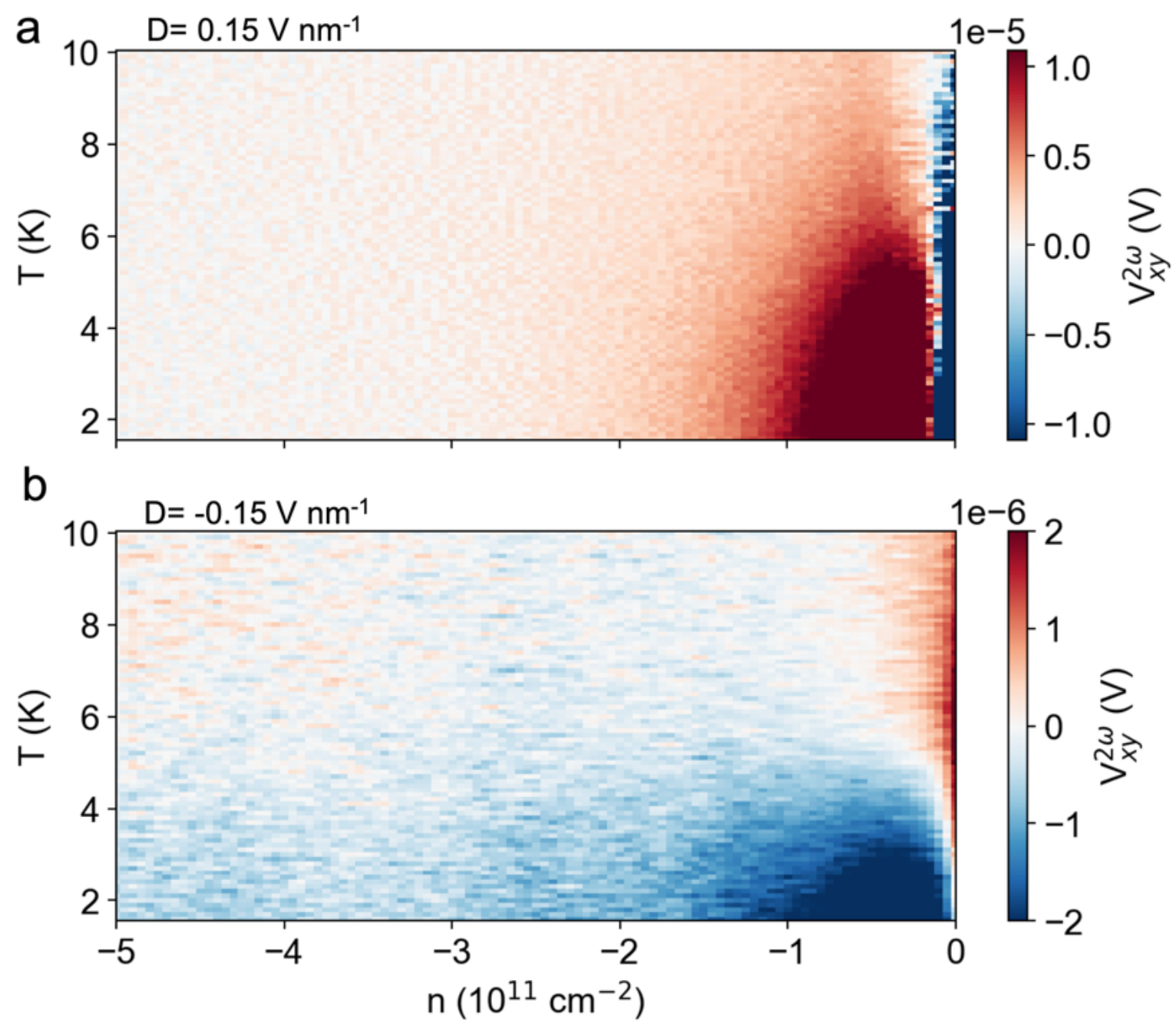

Supplementary Figure 6. Temperature and carrier density dependence of nonlinear Hall voltages in device 2. (**a**) Results at *D*=0.15 (**b**) Results at D=-0.15 V nm^{-1}.

The temperature dependence of nonlinear Hall voltages of two *D*=0.15 and -0.15 V nm^{-1} are presented in Supplementary Figure 6a&b, respectively. Within a fixed plotting range, the giant low-temperature nonlinear signal is rapidly suppressed on the scale of several kelvin, so that it appears to vanish beyond ~8 K. However, when measured separately at each fixed temperature with appropriate sensitivity, a weaker residual nonlinear signal remains detectable at higher temperatures.

Supplementary Section VII: Nonlinear transverse and longitudinal response

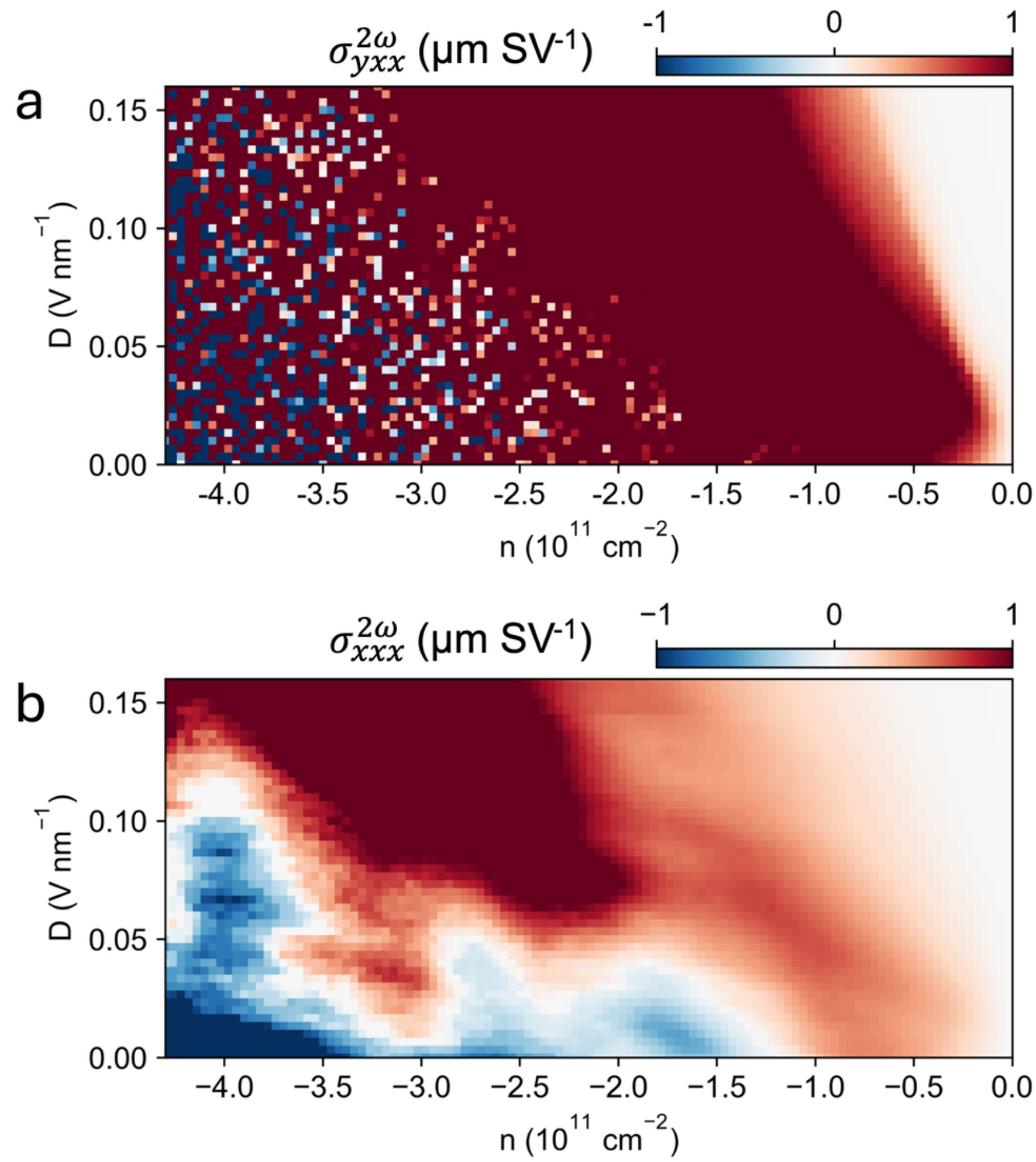

Supplementary Figure 7. Nonlinear transverse and longitudinal response. Transverse (a) and longitudinal (b) second-order response in BBG, measured in device 3.

To examine the tensorial nature of the nonlinear response, we measured both transverse and longitudinal second-order signals in an additional device. As shown in Supplementary Figure 7, the longitudinal response is finite over a broad region of the phase space, while the transverse nonlinear Hall response is more localized near the symmetry-broken regime. These measurements confirm that the nonlinear

response is not restricted to a single Hall-like channel. However, the Hall-bar geometry does not allow a full reconstruction of the rank-3 nonlinear conductivity tensor. Moreover, the longitudinal second-harmonic voltage is more susceptible to Joule-heating-induced thermoelectric contributions. Therefore, in the main text we focus on the transverse nonlinear Hall component, which provides the most phase-sensitive and experimentally robust signature in our device.

Supplementary Section VIII: FFT result of quantum oscillations in an additional device.

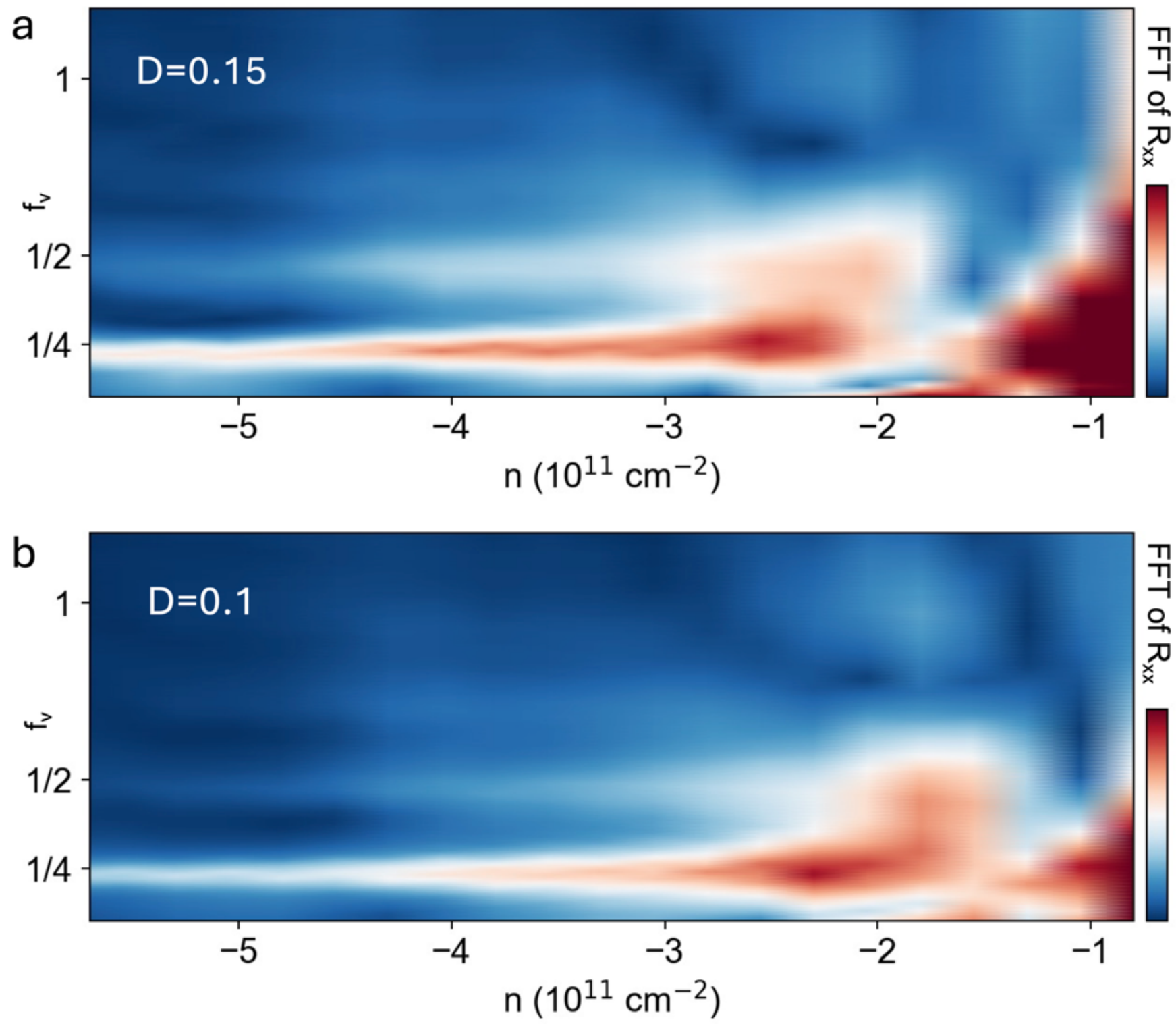

Supplementary Figure 8. FFT colormap as the function of carrier density n at different D-field conditions. a, Results at D= 0.1 V nm^{-1} b, Results at D= 0.1 V nm^{-1}. Results are acquired in device 3.

Supplementary Section IX: Linear Hall measurement results at PIP2 state

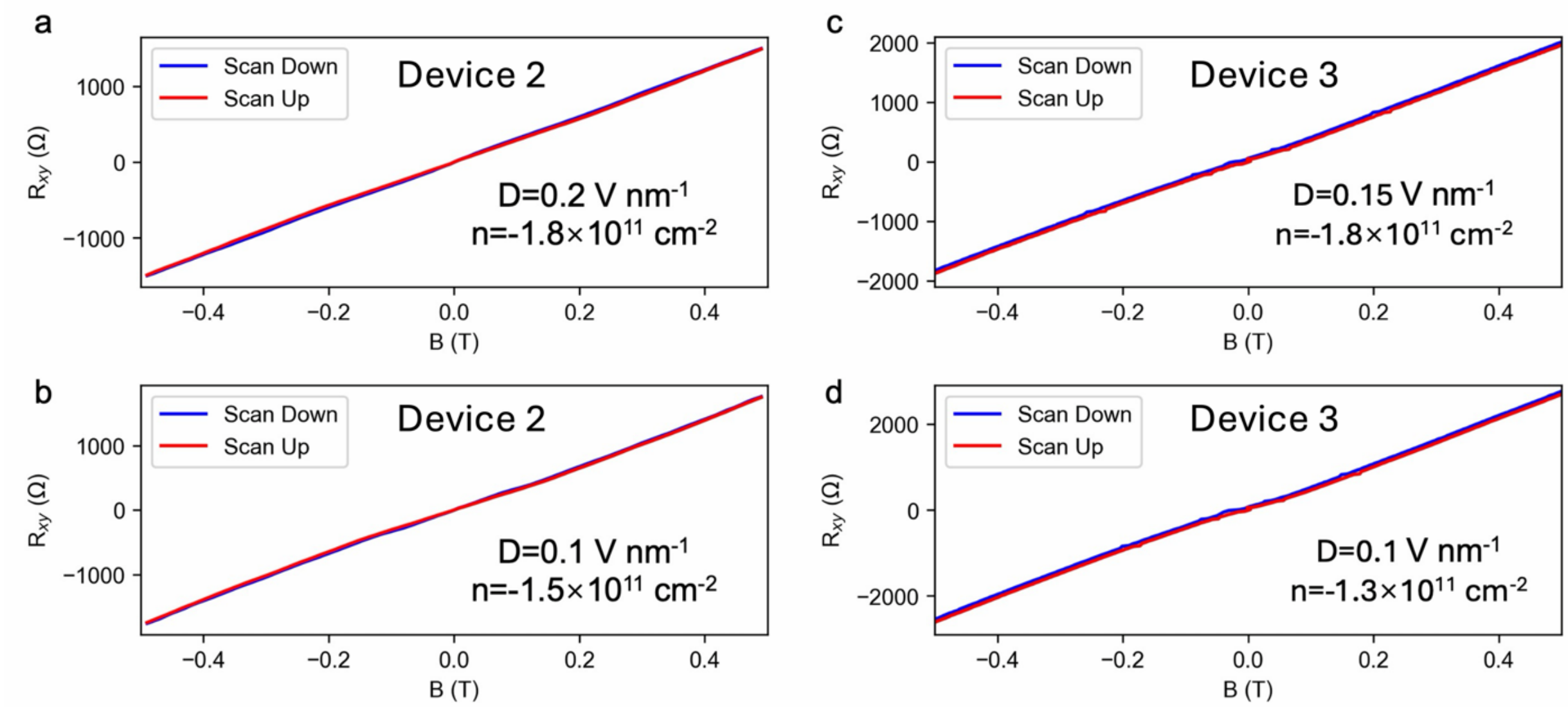

Supplementary Figure 9. Linear Hall measurement results at PIP2 state. Linear Hall measurement results at PIP2 states in device 2 (a&b) and device 3 (c&d), respectively. No obvious AHE is observed in our devices.

Supplementary Section X: Determination of the theoretical phase diagram

Low-Energy Effective Hamiltonian:

The single-particle electronic structure of Bernal-stacked bilayer graphene (BBG) is described using the Slonczewski-Weiss-McClure (SWMcC) parametrization[1-3]. In second-quantized notation, the non-interacting Hamiltonian $\mathcal{H}_0$ is given by,

$$H_0 = \sum_{\boldsymbol{k},\xi,s} \Psi^{\dagger}_{\boldsymbol{k},\xi,s} \mathcal{H}_0^{\xi}(\boldsymbol{k}) \Psi_{\boldsymbol{k},\xi,s}$$

where $\Psi_{\mathbf{k},\xi,s} = (c_{A1}, c_{B1}, c_{A2}, c_{B2})^T$ is the basis spinor for valley $\xi = \pm 1$ and spin $s = \uparrow, \downarrow$. The matrix $\mathcal{H}_0^{\xi}(\mathbf{k})$ is defined as:

$$\mathcal{H}_0^{\xi}(\mathbf{k}) = \begin{pmatrix} -\Delta/2 & \xi v_0 k_- & -\xi v_4 k_- & \xi v_3 k_+ \\ \xi v_0 k_+ & -\Delta/2 & \gamma_1 & -\xi v_4 k_- \\ -\xi v_4 k_+ & \gamma_1 & \Delta/2 & \xi v_0 k_- \\ \xi v_3 k_- & -\xi v_4 k_+ & \xi v_0 k_+ & \Delta/2 \end{pmatrix}$$

where $k_{\pm} = k_x \pm i k_y$ and the velocities are given by $v_i = \frac{\sqrt{3}a}{2\hbar}\gamma_i$ with the γ_i's representing the $k.p$ hopping parameters. Δ represents the displacement field (D) induced layer asymmetry potential. We have considered $\gamma_0 = 3.16$, $\gamma_1 = 0.381$, $\gamma_3 = 0.38$, $\gamma_4 = 0.14$ eV.

cRPA Determination of Interaction Parameters

To account for the high-frequency dielectric environment provided by the hBN encapsulation, we have incorporated the constrained Random Phase Approximation (cRPA) as implemented within VASP[4-6] simulation package. For exchange correlations Perdew Burke Ehrenzoff potentials have been used. The Hilbert space is partitioned into a target "active" subspace comprising the π and π^* bands and a "passive" subspace (Q) including the σ bands and hBN states. The partially screened interaction $W(\omega = 0)$ is defined via the polarization of the passive subspace (χ_Q) as:

$$W = \left[1 - V\chi_Q\right]^{-1} V$$

where V is the bare Coulomb interaction. The interaction parameters U_{spin} and U_{valley} are extracted from the static limit of the screened matrix W_{ijkl}.

U_{spin} is determined by the on-site Hubbard term$W_{ii,ii}$, and found to be 0.53 eV. U_{valley} is derived from the difference between the intra-valley interaction and the inter-valley exchange and found to be 1.02 eV. Due to the large momentum transfer involved in intervalley processes, screening from the hBN environment is less effective at these scales, leading to a larger value.

Mean-Field Self-Consistency calculation:

The mean-field interaction term was defined as

$$H_{int} = -\sum_{k,\xi,s} \Psi^{\dagger}_{k,\xi,s} \left(U_{spin}\langle m_z\rangle s_z + U_{valley}\langle \eta_z\rangle \tau_z\right)\Psi_{k,\xi,s}$$

where s_z and τ_z are the Pauli matrices in spin and valley space. The order parameters are the spin magnetization $m_z = n_\uparrow - n_\downarrow$ and the valley polarization $\eta_z = n_K - n_{K'}$.

The total Hamiltonian $H = H_0 + H_{int}$ is solved self-consistently until the valley polarization gap $\Delta_v = U_{valley}\eta_z$ and the spin polarization gap $\Delta_s = U_{spin}m_z$ is stable within $0.1\ meV$.

Berry Curvature:

From the converged eigenstates $|u_{n,\mathbf{k}}\rangle$, the Berry curvature for band n with energy $\epsilon_{n,\mathbf{k}}$ is computed using the Kubo formula[7]:

$$\Omega_n(k) = -2\mathrm{Im}\sum_{m\neq n}\frac{\langle u_{n,k}|\widehat{v_x}|u_{m,k}\rangle\langle u_{m,k}|\widehat{v_y}|u_{n,k}\rangle}{\left(\epsilon_{n,k} - \epsilon_{m,k}\right)^2}$$

Where, v_x and v_y are the velocity operators.

References:

1 Slonczewski, J. C. & Weiss, P. R. Band Structure of Graphite. *Physical Review* **109**, 272-279 (1958). https://doi.org:10.1103/PhysRev.109.272

2 McClure, J. W. Band Structure of Graphite and de Haas-van Alphen Effect. *Physical Review* **108**, 612-618 (1957). https://doi.org:10.1103/PhysRev.108.612

3 Dresselhaus, M. S. & Dresselhaus, G. Intercalation compounds of graphite. *Advances in Physics* **51**, 1-186 (2002). https://doi.org:10.1080/00018730110113644
4 Kresse, G. & Hafner, J. Ab initio molecular dynamics for liquid metals. *Physical Review B* **47**, 558-561 (1993). https://doi.org:10.1103/PhysRevB.47.558
5 Kresse, G. & Furthmüller, J. Efficient iterative schemes for ab initio total-energy calculations using a plane-wave basis set. *Physical Review B* **54**, 11169-11186 (1996). https://doi.org:10.1103/PhysRevB.54.11169
6 Kresse, G. & Furthmüller, J. Efficiency of ab-initio total energy calculations for metals and semiconductors using a plane-wave basis set. *Computational Materials Science* **6**, 15-50 (1996). https://doi.org:https://doi.org/10.1016/0927-0256(96)00008-0
7 Wang, X., Yates, J. R., Souza, I. & Vanderbilt, D. Ab initio calculation of the anomalous Hall conductivity by Wannier interpolation. *Physical Review B* **74**, 195118 (2006). https://doi.org:10.1103/PhysRevB.74.195118
