# Supplementary material for: Giant nonlinear Hall effect in isospin symmetry broken bilayer graphene
Source: Nat Commun. 2026 Jul 20;17:8856. doi: 10.1038/s41467-026-75919-4 (PMC13500830; doi:10.1038/s41467-026-75919-4)
Supplement: Supplementary file 2 [file 41467_2026_75919_MOESM2_ESM.pdf]

nature portfolio

Peer Review File

Giant Nonlinear Hall effect in Isospin Symmetry Broken Bilayer Graphene

Corresponding Author: Professor Kian Ping Loh

This file contains all reviewer reports in order by version, followed by all author rebuttals in order by version.

Version 0:

Reviewer comments:

Reviewer #1

(Remarks to the Author)
This work studies the nonlinear Hall effect in Bernal bilayer graphene driven by interaction-induced isospin symmetry breaking. The authors find that the nonlinear response no longer follows the conventional quadratic scaling relation with linear conductivity, but instead exhibits an exponential scaling behavior. They attribute this phenomenon to the spontaneous formation of valley polarization. This work is interesting. My comments are as follows.
(1) Why do the authors attribute the nonlinear Hall effect observed here to skew scattering?
(2) The nonlinear Hall response at the electron side is orders of magnitude smaller than that at the hole side, and no PIP2 state is observed. However, the authors do not provide a very clear explanation for this.
(3) The main text presents the scaling relation under different carrier concentrations, while the supplementary materials show similar scaling behavior in another device under various displacement fields. It is recommended to emphasize this in the main text to enhance the universality of this unconventional scaling relation.
(4) The authors compare the magnitude of the nonlinear Hall conductivity in this work with that of other systems such as Moiré-engineered graphene. In comparison to these systems, does the nonlinear Hall conductivity in this work exhibit advantages? The authors need to add relevant discussion.
(5) Some figures lack information. The meaning of each component in the schematic diagram of Fig. 1a is not indicated. The vertical axis in Fig. 3a has no units.
(6) Certain expressions in the manuscript need to be revised. Some symbols and abbreviations are not defined upon their first appearance, such as "NLH". Additionally, there are grammatical issues in some sentences, for example, "These authors contribute equally the work".

Reviewer #2

(Remarks to the Author)
The manuscript by Hao Chen et al. reports a giant nonlinear Hall effect in Bernal bilayer graphene. This work employs a system in which symmetry breaking can be dynamically tuned, establishing spontaneous isospin symmetry breaking as a new mechanism for generating nonlinear quantum transport. The study is of broad interest to the community. However, several key issues, especially regarding the interpretation of the mechanism, need to be addressed before the work can be considered for publication.

1. The authors attribute the observed exponential temperature dependence to a skew scattering mechanism involving thermally activated minority valley populations.
a. Have the authors calculated the temperature dependence of the Berry curvature dipole (BCD)? Is it possible that the exponential response arises from strong temperature-dependent variations in the band structure that lead to a sharply changing BCD?

b, Figure 4 currently only explains the presence or absence of a nonlinear signal in two different states. Is there more direct evidence, such as a quantitative estimate of the skew scattering induced nonlinear conductivity that is comparable with experimental magnitudes, that supports skew scattering as the dominant mechanism?

2. The PIP2 state is described as time-reversal symmetry broken. If so, a nonzero linear Hall effect would generally be

expected at zero magnetic field. Was any such signal observed?

3. In Figure 3, the nonlinear Hall signal persists up to ~70 K, but Supplementary Figure 6 shows the signal vanishing by ~8 K. Please clarify the origin of this discrepancy.

4. The authors cite a recent preprint (ref. 18) that reports a nonlinear Hall conductivity of similar magnitude in Bernal bilayer graphene. However, that work states that the nonlinear conductivity extracted in the Sym4 phase is almost 100 times larger than that in the PIP2 phase, which appears to contradict the current manuscript's claim that the giant nonlinear signal arises exclusively in the PIP2 state. Could the authors comment on this apparent discrepancy? Is it due to different measurement conditions, device structure, or device quality? Clarifying this point would help situate the present findings within the broader context.

5. The authors use quantum oscillation measurements to distinguish between the PIP2 and Sym4 phases. However, both the top and bottom gates in the device are made of graphite, which itself exhibits oscillatory behavior in a magnetic field. The oscillatory density of states in the graphite gates can modulate the device capacitance and, consequently, cause small changes in the carrier density of the bilayer graphene channel as a function of magnetic field. Could this effect influence the extracted oscillation frequencies or the assignment of filling fractions?

6. Figure 1a: Only one of the source/drain electrodes is labeled. Please label both for clarity.

Reviewer #3

(Remarks to the Author)
Report on Manuscript: “Giant Nonlinear Hall Effect in Isospin Symmetry Broken Bilayer Graphene”

Summary:

The authors report a giant nonlinear Hall conductivity of 9.1 $\mu A\ V^{-2}$ in dual-gated Bernal bilayer graphene (BBG), which they attribute to spontaneous isospin symmetry breaking under a perpendicular displacement field. Using quantum oscillation measurements, they identify a partially isospin-polarized (PIP2) state as the origin of this effect. Furthermore, they report an anomalous exponential scaling of the nonlinear conductivity with the linear Drude conductivity at low temperatures. The topic is highly timely, and the experimental observation of a giant nonlinear response in clean BBG is intrinsically interesting and relevant to the community.

However, I have several major concerns that must be addressed before the manuscript can be considered for publication. These concerns primarily stem from the physical implications of valley polarization acting as a time-reversal-symmetry-breaking mechanism.

1. Absence of Linear Anomalous Hall Data:
The central interpretive claim of the paper is that the PIP2 state is a valley-polarized state that spontaneously breaks macroscopic time-reversal symmetry (TRS). If TRS is indeed broken, and the Berry curvature flux approaches 2π as claimed in Figure 4b, one would generically expect a finite linear anomalous Hall conductivity (σ_{xy}) in the PIP2 regime. However, the authors neither measure nor discuss the linear Hall conductivity anywhere in the manuscript. To substantiate their claims regarding the symmetry-breaking nature of the PIP2 state, the authors must present the linear Hall conductivity data in this regime and explicitly discuss its magnitude and behavior in the context of broken TRS.

2. Incomplete Treatment of the Nonlinear Conductivity Tensor:
The nonlinear conductivity tensor is a rank-3 tensor. Depending on the exact symmetry of the system, it generally possesses multiple independent components in 2D. The authors utilize a Hall-bar geometry but only measure and report a single transverse component, $\sigma^{2\omega}_{yxx}$. To provide a complete picture of the nonlinear response, the authors should, at a minimum, discuss the expected magnitude of the longitudinal nonlinear response ($\sigma^{2\omega}_{xxx}$) relative to the transverse (Hall) component and explain whether their experimental geometry allows them to extract or bound this value.

3. Incomplete Scaling Analysis and Recent Theoretical Developments:
The authors' interpretation heavily relies on the Berry curvature dipole. I would like to draw the authors' attention to recent theoretical progress on nonlinear responses in materials with broken time-reversal symmetry (see, for example, arXiv:2410.04995). In TRS-broken systems, there are numerous distinct contributions to the nonlinear Hall effect beyond the standard Berry curvature dipole. The authors should perform a more comprehensive scaling-law analysis that accounts for these TRS-breaking-specific contributions. This expanded analysis would significantly improve the theoretical understanding of their anomalous scaling observation and strengthen the manuscript's conclusions.

With these improvements, I will be in a better position to recommend publication.

Version 1:

Reviewer comments:

Reviewer #1

(Remarks to the Author)
The authors have addressed all the concerns and claricattions I raised. Now, I recommend the publication of this work.

Reviewer #2

(Remarks to the Author)
All previous questions have been addressed satisfactorily. I have only one small remaining concern.

In the Abstract, the concluding sentence states that the effect is "governed by the interplay of quantum geometry and strong electron correlations." The mechanism advanced in this work is skew scattering. While skew scattering can be connected to quantum geometry, the current phrasing is misleading: it suggests that an intrinsic quantum geometric mechanism, such as the Berry curvature dipole or quantum metric, is directly responsible for the observed signal. The authors should revise this sentence and any related passages to explicitly identify skew scattering as the dominant transport mechanism.

With this revision, I recommend publication in Nature Communications.

Reviewer #3

(Remarks to the Author)
The authors clarified almost all the issues. The theoretical explanation a little bit hand waving but can be understood. This is because the non-linear behavior cannot be explained using semi-classical transport argument.

Open Access This Peer Review File is licensed under a Creative Commons Attribution 4.0 International License, which permits use, sharing, adaptation, distribution and reproduction in any medium or format, as long as you give appropriate credit to the original author(s) and the source, provide a link to the Creative Commons license, and indicate if changes were made.
In cases where reviewers are anonymous, credit should be given to 'Anonymous Referee' and the source.
The images or other third party material in this Peer Review File are included in the article's Creative Commons license, unless indicated otherwise in a credit line to the material. If material is not included in the article's Creative Commons license and your intended use is not permitted by statutory regulation or exceeds the permitted use, you will need to obtain permission directly from the copyright holder.
To view a copy of this license, visit https://creativecommons.org/licenses/by/4.0/

Point-by-point responses to reviewers for Manuscript **NCOMMS-26-008629-T**

Reviewer #1 (Remarks to the Author):

This work studies the nonlinear Hall effect in Bernal bilayer graphene driven by interaction-induced isospin symmetry breaking. The authors find that the nonlinear response no longer follows the conventional quadratic scaling relation with linear conductivity, but instead exhibits an exponential scaling behavior. They attribute this phenomenon to the spontaneous formation of valley polarization. This work is interesting. My comments are as follows.

Response: We thank the reviewer for recognizing the novelty of our work. Below we reply to the reviewer’s constructive questions in detail.

(1) Why do the authors attribute the nonlinear Hall effect observed here to skew scattering?

Response: We thank the reviewer for this important question. Our data do not by themselves constitute a full microscopic proof that skew scattering is the unique mechanism. Rather, they indicate that the observed nonlinear Hall response is most consistent with a skew-scattering-assisted scenario tied to the interaction-driven valley-polarized PIP2 state. Specifically, the nonlinear response increases abruptly upon entering PIP2 state and is strongly suppressed once the system returns to Sym4 state, which links the effect to valley polarization rather than to a generic inversion-breaking band-geometric response. In addition, the low-temperature exponential scaling relation is difficult to reconcile with a purely intrinsic BCD- or quantum-metric-dipole picture, but arises naturally if asymmetric scattering is amplified by thermally activated minority-valley occupation.

We therefore interpret our results as supporting skew scattering as the leading candidate mechanism in the PIP2 regime, while acknowledging that a complete microscopic disentangling of all allowed second-order contributions remains beyond the scope of the present work.

(2) The nonlinear Hall response at the electron side is orders of magnitude smaller

than that at the hole side, and no PIP2 state is observed. However, the authors do not provide a very clear explanation for this.

Response: We thank the reviewer for raising this point. We agree that the electron–hole asymmetry deserves a clearer explanation. In fact, such asymmetry is commonly observed in BBG and is a natural consequence of its band structure and interaction physics, rather than an unusual feature of the devices in this manuscript.

In our measurements, the key experimental fact is that the giant nonlinear Hall signal tracks the PIP_2 phase one-to-one**.** On the hole side, FFT analysis clearly reveals the $f_v = 1/2$ PIP_2 state, and the nonlinear Hall conductivity is strongly enhanced in the same carrier density/D-field window. By contrast, on the electron side, the FFT shows only the $f_v = 1/4$ Sym_4 state and no PIP_2 signature, and correspondingly the nonlinear Hall response is suppressed by orders of magnitude. Thus, the simplest explanation is that the nonlinear Hall signal is weak on the electron side because the underlying symmetry-broken state that supports the giant response does not develop there in our accessible parameter range.

This electron–hole asymmetry is well documented experimentally in BLG. Early infrared spectroscopy already showed a significant asymmetry between electron and hole doping in bilayer graphene [Li, Z. Q., et al., (2009). Physical Review Letters, 102(3), 037403.], tracing it to an intrinsic asymmetry between the valence and conduction bands. Shubnikov–de Haas studies further found that the hole effective mass is about 20–30% larger than the electron effective mass [Zou, K., et al.,(2011). Physical Review B, 84(8), 085408.], again directly demonstrating electron–hole asymmetry in BLG band structure. Notably, asymmetry is also seen in the correlated(isospin-symmetry-broken) regime of BLG. de la Barrera et al. show that in BBG the hole side exhibits stronger and more interaction-driven features than the electron side: two prominent hole-side compressibility peaks (A, B) versus only a broad electron-side shoulder (C), with the electron-side parent state much less evident at zero field [de la Barrera, et al., (2022). Nature Physics, 18(7), 771-775.]. Likewise, another work [Zhou, H., et al., (2022). Science, 375(6582), 774-778.] on BBG focused on the displacement-field-induced correlated transitions on the hole-doped side, where trigonal warping and the van Hove structure generate rich isospin transitions. These prior results support the view that symmetry-broken metallic phases in BLG often appear much more prominently on one carrier side than the other.

Theoretically, this asymmetry is expected from the standard Slonczewski–Weiss–McClure description of Bernal BLG. As reviewed by McCann and Koshino [McCann, E., & Koshino, M. (2013). Reports on Progress in physics, 76(5), 056503.], BLG contains several intrinsic sources of electron–hole asymmetry, especially the skew interlayer hopping γ_4, the dimer/non-dimer site energy difference Δ', and also next-nearest-neighbour hopping terms. These terms make the conduction and valence bands quantitatively inequivalent. In addition, trigonal warping and the associated Lifshitz transition strongly reshape the low-energy Fermi surfaces. The valence band is often found to develop off-center pockets and topology changes more readily, whereas the conduction band evolves differently under the same displacement field. Because the formation of PIP_2 depends sensitively on the detailed Fermi-surface topology, density of states, and exchange energy balance, even modest single-particle electron–hole asymmetry can produce a strong asymmetry in the stability window of the correlated phase.

Therefore, our interpretation is that the much smaller nonlinear Hall response on the electron side is a direct consequence of the absence of the PIP_2 state there, and that this asymmetry is consistent with the known intrinsic and interaction-sensitive electron–hole asymmetry of Bernal bilayer graphene. We will revise the manuscript to make this point more explicit and to cite the relevant BLG literature.

(3) The main text presents the scaling relation under different carrier concentrations, while the supplementary materials show similar scaling behavior in another device under various displacement fields. It is recommended to emphasize this in the main text to enhance the universality of this unconventional scaling relation.

Response: We thank the reviewer for this helpful suggestion. We agree that the universality of the unconventional scaling relation deserves stronger emphasis in the main text. In the present work (with an additional device 3 data), we demonstrate this scaling relation in two complementary ways: first, in Figure 3d and Supplementary Figure 5b, by varying carrier density at fixed displacement field; and second, in the Supplementary Figure 5a, by varying displacement field at fixed carrier density. The observation of the same scaling form under both tuning parameters, and across different devices, indicates that this unconventional scaling is a robust and intrinsic

characteristic of the nonlinear transport in the relevant symmetry-broken phase, rather than a parameter-specific or device-specific effect. We will revise the main text accordingly to highlight this universality more clearly.

(4) The authors compare the magnitude of the nonlinear Hall conductivity in this work with that of other systems such as Moiré-engineered graphene. In comparison to these systems, does the nonlinear Hall conductivity in this work exhibit advantages? The authors need to add relevant discussion.

Response: We thank the reviewer for this helpful comment. We note that this comparison is already included in the manuscript. As stated in the main text, “the nonlinear Hall conductivity reaches 9.1 μm SV^{-1}. This magnitude exceeds that of pristine WTe_2 and $MoTe_2$ crystals by several orders of magnitude and is on par with the Moiré-engineered graphene devices.” We further note in the conclusion that the observed value is “orders of magnitude larger than that of pristine inversion-broken crystals … and comparable to values reported in engineered moiré systems.”

Compared with moiré-engineered graphene platforms, the main advantage of the present work is that a similarly giant nonlinear Hall conductivity is achieved in Bernal bilayer graphene without moiré engineering or mechanical strain, but instead through electrically tunable spontaneous isospin symmetry breaking. This provides a simpler and more intrinsic platform, where the nonlinear response can be controlled continuously by carrier density and displacement field, and directly linked to the emergence of the PIP_2 isospin-polarized state. In this sense, the advantage of our system is not necessarily a larger absolute magnitude than moiré devices, but rather that it realizes a comparably strong nonlinear Hall response in a non-moiré, electrically programmable, and conceptually distinct correlated mechanism.

(5) Some figures lack information. The meaning of each component in the schematic diagram of Fig. 1a is not indicated. The vertical axis in Fig. 3a has no units.

Response: We thank the reviewer for pointing out these details. We have added a detailed description of the device schematic in Fig. 1a caption and specified vertical axis unit in Fig. 3a.

(6) Certain expressions in the manuscript need to be revised. Some symbols and abbreviations are not defined upon their first appearance, such as "NLH". Additionally, there are grammatical issues in some sentences, for example, "These authors contribute equally the work".

Response: We thank the reviewer for careful check on the manuscript. We have accordingly revised the undefined abbreviations in the manuscript and corrected the relevant grammatical errors throughout the text.

Reviewer #2 (Remarks to the Author):

The manuscript by Hao Chen et al. reports a giant nonlinear Hall effect in Bernal bilayer graphene. This work employs a system in which symmetry breaking can be dynamically tuned, establishing spontaneous isospin symmetry breaking as a new mechanism for generating nonlinear quantum transport. The study is of broad interest to the community. However, several key issues, especially regarding the interpretation of the mechanism, need to be addressed before the work can be considered for publication.

Response: We thank the reviewer for acknowledging the novelty and value of our work. We also thank the reviewer for constructive comments and suggestions which we address point by point as below.

1. The authors attribute the observed exponential temperature dependence to a skew scattering mechanism involving thermally activated minority valley populations.
a. Have the authors calculated the temperature dependence of the Berry curvature dipole (BCD)? Is it possible that the exponential response arises from strong temperature-dependent variations in the band structure that lead to a sharply changing BCD?

Response: We thank the reviewer for this important question. In our current theoretical modelling we did not focus on the Berry curvature dipole (BCD) mechanism because within the C_{3v} point group symmetry relevant to our device, the in-plane BCD is expected to be strongly suppressed.

Pristine Bernal-stacked (AB) bilayer graphene possesses D_{3d} point group symmetry. While out-of-plane field breaks inversion symmetry, the in-plane rotational symmetry remains intact. Furthermore, the correlation driven phase transition in our system, governed by the interaction parameters U_{valley} and U_{spin} operates primarily in the internal isospin space. While these interactions spontaneously break time-reversal symmetry and valley degeneracy to stabilize the isospin symmetry broken state, they do not introduce a resolved uniaxial lattice distortion or nematic spatial symmetry breaking. Under these assumptions, a sizable macroscopic in-plane BCD is not expected.

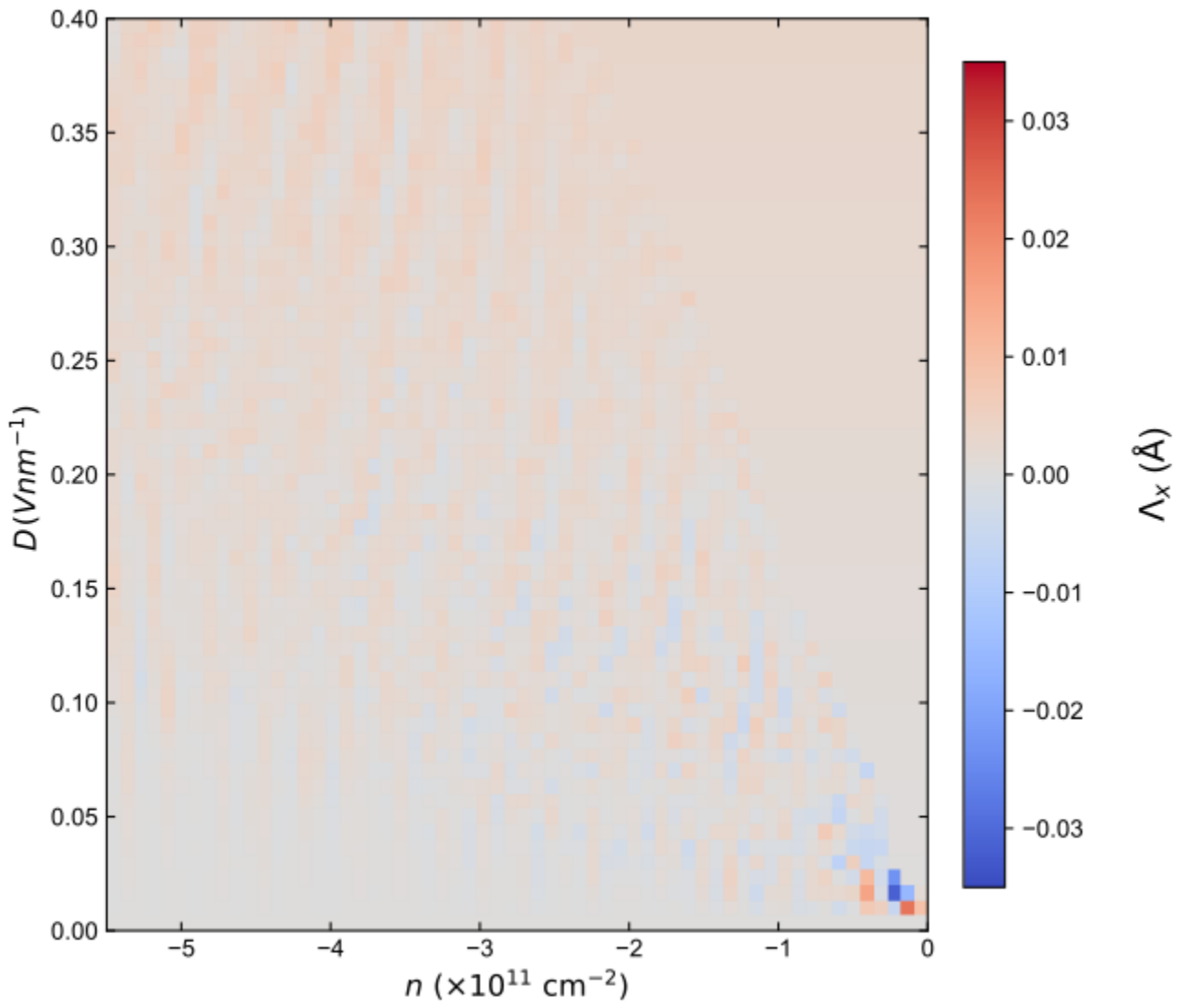

Figure R1. Berry Curvature Dipole (Λ_x) distribution of BLG in (n, D) space.

To support this point, we numerically evaluated the BCD for our system over the relevant phase space in Figure R1. In the following figure we have plotted $D_x = \int \frac{d^2k}{(2\pi)^2} f_0(k) \frac{\partial \Omega_z}{\partial k_x}$, where Ω_z is the z component of the Berry curvature and f_0 is the Fermi Dirac distribution, for the entire phase space of (n, D). Matching with the symmetry argument we found the BCD component to be vanishingly small for the isospin symmetry broken region. The residual value arises from numerical discretization of the k-grid and does not represent a physically significant dipole. In

our calculation the BCD scale is 2 to 3 orders of magnitude smaller than that of WTe_2 (a prototypical example of BCD activated NLH transport), and is far too small to account for the experimentally observed giant nonlinear Hall effect. Therefore within our modelling framework and symmetry assumptions, the BCD contribution is negligible and unlikely to explain the observed nonlinear effect.

b, Figure 4 currently only explains the presence or absence of a nonlinear signal in two different states. Is there more direct evidence, such as a quantitative estimate of the skew scattering induced nonlinear conductivity that is comparable with experimental magnitudes, that supports skew scattering as the dominant mechanism?

Response: We thank the reviewer for the question. To show that the experimentally observed nonlinear Hall signal is compatible with the skew-scattering mechanism in the PIP_2 state, we provide an order-of-magnitude estimate on experimentally constrained and phenomenological parameters.

For an electric field $\vec{E}$ along x direction, using relaxation time approximation The Boltzmann transport equation can be written as,

$$(-eE_x)\frac{\partial f}{\partial k_x} = -\frac{f - f_0}{\tau}$$

Using a perturbative expansion for the distribution function, $f = f_0 + f^{(1)} + f^{(2)} + \cdots$, we get up to second order,

$$-eE_x\frac{\partial}{\partial k_x}\left[f_0 + f^{(1)} + f^{(2)}\right] = -\frac{f^{(1)} + f^{(2)}}{\tau}$$

Collecting the same order terms of electric field,

1st order correction : $-eE_x\frac{\partial f_0}{\partial k_x} = -\frac{f^{(1)}}{\tau} \Rightarrow f^{(1)} = e\,\tau E_x\frac{\partial f_0}{\partial k_x}$

2nd order correction : $-eE_x\frac{\partial f^{(1)}}{\partial k_x} = -\frac{f^{(2)}}{\tau} \Rightarrow f^{(2)} \approx e\tau E_x\frac{f^{(1)}}{K_F} = \frac{\mu E_x}{v_F}f^{(1)}$

In the last line, assuming that the variation of $f^{(1)}$ occurs over the Fermi momentum scale, the derivative, $\frac{\partial f^{(1)}}{\partial k_x}$ has been approximated by $\frac{f^{(1)}}{K_F}$ and the Drude definition of $\mu = \frac{e\tau}{m^*}$ have been used.

The second order response to the field $f^{(2)}$ will generate the transverse shift in the symmetry broken state, due to asymmetric scattering. The net second order

transverse response in the isospin symmetry broken gated bilayer graphene due to skew scattering can now be phenomenologically written as proportional to both the population imbalance between K and K' valleys, $\eta = \frac{n_k - n_{k'}}{n_k + n_{k'}}$ and the macroscopic skew scattering Hall angle, θ_{skew}, defined as the ratio of the asymmetric to symmetric scattering contributions or physically measuring average transverse deflection per scattering event.

Therefore,

$$J_{y,\ skew}^{(2\omega)} = -e \int v \times \left[\eta \theta_{skew} f^{(2)}\right] d^2k \approx \eta \theta_{skew} \frac{\mu E_x}{v_F} \times \left[-e \int v f^{(1)} d^2k\right] \approx \eta \theta_{skew} \frac{\mu E_x}{v_F} \sigma_{xx} E_x$$

Writing the mobility as $\mu = \frac{\sigma_{xx}}{ne}$, we arrive at the scaling law for the second order signal,

$$\sigma_{yxx,\ skew}^{(2\omega)} \approx \eta \theta_{skew} (1/nev_F) \sigma_{xx}^2$$

In order to get an order of estimate for the transverse second order response due to skew scattering we can put in the values of the right side parameters as follows,

- For valley population imbalance, η, a value of 1 or 0 implies completely isospin symmetry broken state or symmetric state. We choose a typical value of $\eta = 0.8$
- The skew scattering angle, θ_{skew}, is a macroscopic phenomenological parameter which can be reasonably bounded based on previous studies. Both theoretical transport calculations for 2D electron gases with broken symmetries (Milletarì et al., Phys. Rev. B 82, 2010) and experimental extractions in high-conductivity metals (Hou et al., Appl. Phys. Lett. 102, 2013) places the skew-scattering transport angle in the order-of-magnitude boundary of 10^{-3} to 10^{-2} (Nagaosa et al., Rev. Mod. Phys. 82, 2010). For our calculation we take a moderate value , $\theta_{skew} = 5 \times 10^{-3}$
- The carrier density $n = 2 \times 10^{15}\ m^{-2}$ and the Fermi velocity, $v_F = 1.7 \times 10^2\ mS^{-1}$ is obtained from the k.p Hamiltonian described in methodology part in the supplementary information.
- Longitudinal conductivity, $\sigma_{xx} = 2.2 \times 10^{-3}\ S$ has been retrieved from the experimentally measured value at the particular doping concentration.

Using representative values for the valley population imbalance, skew-scattering angle, carrier density, Fermi velocity, and longitudinal conductivity, we obtain an estimate for the nonlinear Hall response $\sigma^{(2\omega)}_{yxx,\ skew} \approx 0.3\ \mu m\ SV^{-1}$, which is of the same order of magnitude as the experimental value shown in Figure 2a.

While the phenomenological approach described above yields agreement with our experimental observations, a fully microscopic calculation of the nonlinear conductivity tensor from first principles remains out of scope for the current study. Evaluating the exact asymmetric scattering transition rates $\mathcal{W}_{\boldsymbol{kk'}}$ requires solving the full T-matrix or third-order Born approximation using microscopic details of the physical disorder potential. Our point here is that once valley polarization develops in the PIP2 state, an asymmetric-scattering contribution becomes natural on symmetry grounds and can yield a nonlinear Hall response of the observed scale. The extracted activation behavior and scaling relations therefore support the relevance of such a mechanism, while a fully microscopic evaluation remains an important subject for future work.

2. The PIP2 state is described as time-reversal symmetry broken. If so, a nonzero linear Hall effect would generally be expected at zero magnetic field. Was any such signal observed?

Response: We thank the reviewer for this important question. In our measurements, we did not resolve a finite zero-field linear Hall signal in the PIP_2 regime within our experimental sensitivity.

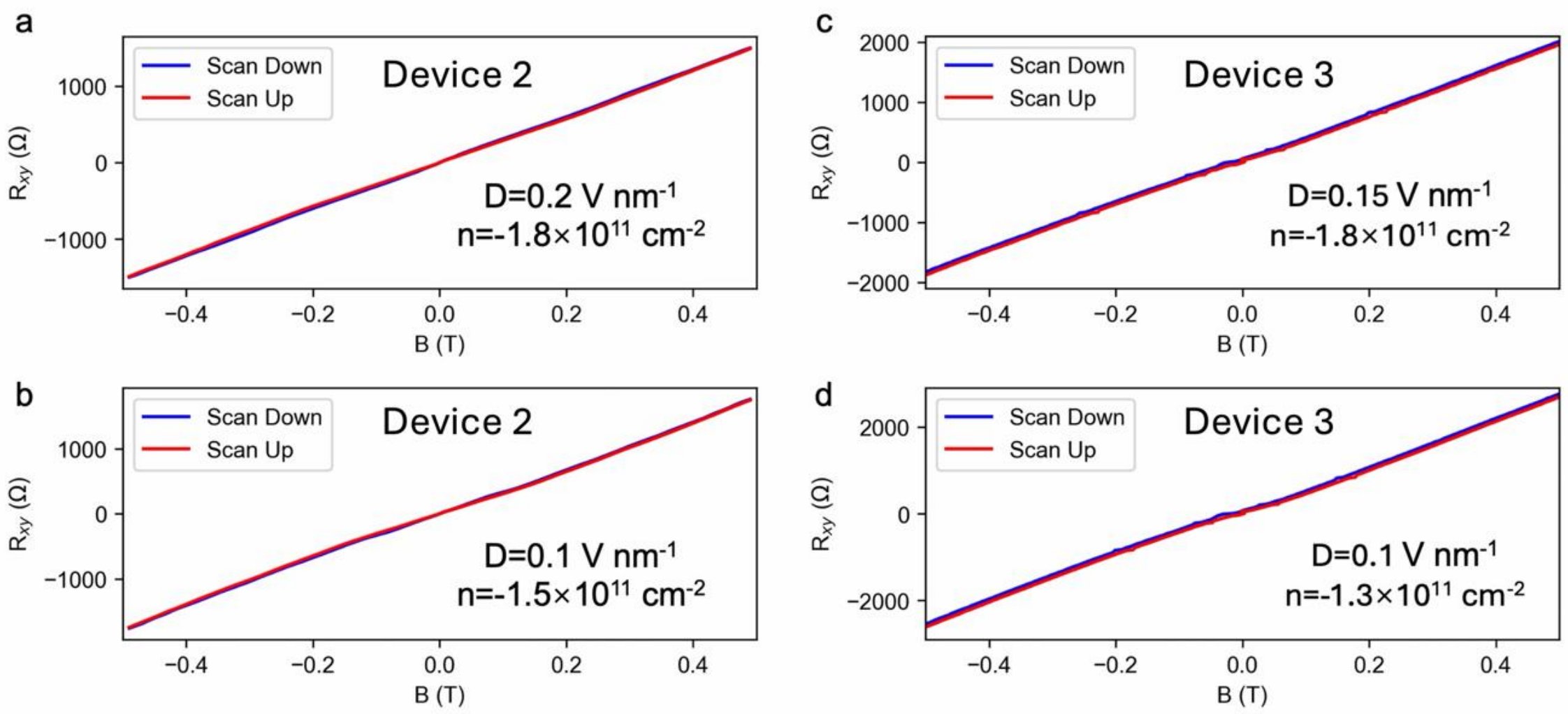

Figure R2. Anomalous Hall measurement results at PIP_2 states in device 2 (a&b) and device 3 (c&d), respectively. Corresponding (n, D) conditions are denoted in the insets.

We would like to emphasize that, although the PIP_2 state is discussed as time-reversal-symmetry broken, this does not by itself guarantee a readily observable linear anomalous Hall effect. Time-reversal-symmetry breaking is a necessary condition for intrinsic zero-field AHE, but the experimentally measurable linear Hall response is set by the net first-order anomalous Hall conductivity, which depends on the momentum-integrated Berry-curvature contribution and can remain very small because of partial cancellation. By contrast, the nonlinear Hall effect (in this context) is a second-order response and is much more sensitive to the detailed Berry-curvature distribution and symmetry lowering near the Fermi surface. As a result, a sizeable NLHE can coexist with an undetectably small linear Hall signal.

A similar distinction has been noted in Ref 18, where the authors reported a nearly vanishing anomalous Hall effect in the same parameter regime while still observing a nonlinear Hall response. We will revise the manuscript to clarify that, although the PIP_2 state is discussed as time-reversal-symmetry broken, in our device this is manifested much more clearly in the nonlinear Hall channel than in the linear channel and no discernible zero-field linear Hall effect was resolved within our measurement sensitivity.

3. In Figure 3, the nonlinear Hall signal persists up to ~70 K, but Supplementary Figure 6 shows the signal vanishing by ~8 K. Please clarify the origin of this discrepancy.

Response: We thank the reviewer for pointing out this ambiguity. There is no actual inconsistency between Figure 3 and Supplementary Figure 6, but we agree that the current presentation is potentially misleading. The key point is that the giant low-temperature enhancement associated with the PIP_2 regime becomes strongly suppressed on the scale of several kelvin, whereas a much weaker residual nonlinear signal can still be tracked to higher temperatures when the data are measured and plotted with temperature-specific sensitivity.

More specifically, in Supplementary Figure 6 the data are displayed within a fixed signal range, so the rapid suppression of the low-temperature enhanced contribution makes the signal appear to vanish around ~8 K. By contrast, in Figure 3 the nonlinear Hall signal at each temperature point was measured and analysed individually, allowing the weaker high-temperature tail to be resolved over a much broader temperature range. In this sense, Supplementary Figure 6 highlights the disappearance of the giant low-temperature contribution, whereas Figure 3 allows one to follow the residual nonlinear response to substantially higher temperature.

We will revise both the manuscript and the Supplementary Information to make this distinction explicit and avoid the misleading impression that the signal disappears abruptly at ~8 K. In particular, we will clarify that the giant enhancement tied to the low-temperature symmetry-broken regime is lost on the scale of several kelvin, while a weaker residual nonlinear background remains measurable at higher temperatures.

4. The authors cite a recent preprint (ref. 18) that reports a nonlinear Hall conductivity of similar magnitude in Bernal bilayer graphene. However, that work states that the nonlinear conductivity extracted in the Sym4 phase is almost 100 times larger than that in the PIP2 phase, which appears to contradict the current manuscript's claim that the giant nonlinear signal arises exclusively in the PIP2 state. Could the authors comment on this apparent discrepancy? Is it due to different measurement conditions, device structure, or device quality? Clarifying this point would help situate the present findings within the broader context.

Response: We thank the reviewer for this important comment. We agree that the recent preprint (Ref. 18) provides a useful point of comparison. As the reviewer notes, that work reports a finite nonlinear response in both the PIP_2 and Sym_4 phases, and further finds that the extracted nonlinear tensor in the Sym_4 phase can be substantially larger than that in PIP_2. At the same time, the authors themselves emphasize that this behavior is unexpected: in their analysis, the Sym_4 phase also exhibits a finite nonlinear response, while none of the known microscopic mechanisms can account for the observed magnitude in either PIP_2 or Sym_4. We therefore do not view their observation as establishing a universal hierarchy between the two phases, but rather as showing that the nonlinear response in Bernal bilayer graphene can be highly

device- and measurement-dependent in a regime that is not yet fully understood microscopically.

In our device, the giant and robust nonlinear Hall signal is resolved most clearly in the PIP_2 regime. By contrast, in the Sym_4 region the measured second-harmonic Hall voltage becomes much smaller and is close to the sensitivity floor of our setup, such that the extracted $\sigma_{yxx}^{2\omega}$ becomes noticeably noisier and can fluctuate around zero in the (n, D) map. For this reason, our data do not allow a comparably comprehensive analysis of the Sym_4 response. The apparent difference from Ref. 18 may therefore arise, at least in part, from differences in measurement sensitivity, signal-to-noise floor and device-specific conditions such as disorder, misalignment and screening. At present, however, these remain plausible contributing factors rather than firm conclusions that can be established from the existing data. We will revise the manuscript to clarify that our main conclusion is that the **dominant and reliably resolved giant nonlinear Hall signal in our device appears in the PIP_2 regime**, while the response in Sym_4 is too weak in our measurements to support a similarly strong claim.

5. The authors use quantum oscillation measurements to distinguish between the PIP2 and Sym4 phases. However, both the top and bottom gates in the device are made of graphite, which itself exhibits oscillatory behavior in a magnetic field. The oscillatory density of states in the graphite gates can modulate the device capacitance and, consequently, cause small changes in the carrier density of the bilayer graphene channel as a function of magnetic field. Could this effect influence the extracted oscillation frequencies or the assignment of filling fractions?

Response: We thank the reviewer for this thoughtful comment. We agree that, in principle, oscillations in the density of states of graphite gates can modulate the device capacitance and thereby induce small magnetic-field-dependent density variations in the bilayer graphene channel. Such an effect has indeed been reported in graphite-gated devices [Zhu et al., (2021) *Physical Review Letters*, *127*(24), 247702]. Importantly, however, the previously reported graphite-gate-induced oscillation was observed in devices employing very thin few-layer graphite gates, especially 1–3 nm

thick graphite (around 3 to 9 layers) together with a ultrathin (~5nm) hBN layer to maximize capacitive coupling. The same paper further showed that the bottom-gate-induced oscillations (with hBN thickness>25nm) are negligible.

In contrast, in our experiment we intentionally choose thicker graphite films (>7 layers) and thicker hBN layer (> 30nm) in our devices (please refer to Figure R3 for device optical image). Therefore the gate-induced capacitance modulation is expected to be much weaker in such condition. We do not expect the graphite-gate DOS oscillation effect to make a significant contribution to the extracted oscillation frequencies or to the assignment of filling fractions in our device. We therefore believe that the phase identification is robust against any small residual gate-induced capacitance modulation. To avoid confusion, we will add a clarifying statement in the revised manuscript noting that, although such effects have been reported in graphite-gated structures, those experiments were performed in the thin-graphite limit, whereas our device uses substantially thicker graphite gates (>7 layers), for which the influence on the extracted oscillation frequencies and filling-factor assignment should be much smaller.

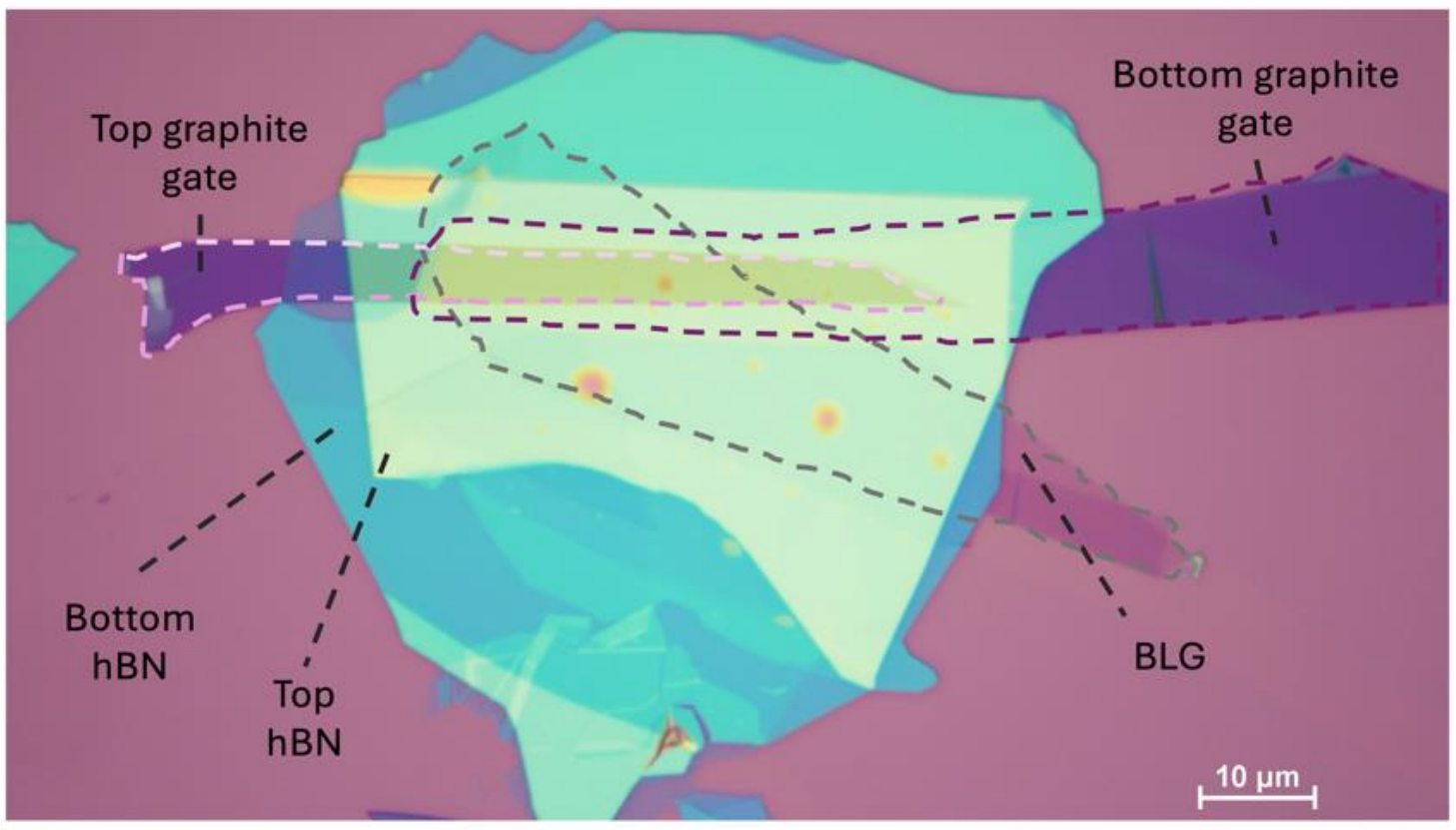

Figure R3. Optical image of the device. Top and bottom graphite gates, hBN layers and BLG are marked accordingly.

6. Figure 1a: Only one of the source/drain electrodes is labeled. Please label both for clarity.

Response: We thank the reviewer for pointing this out. We have labeled both electrodes in Figure 1 accordingly.

Reviewer #3 (Remarks to the Author):

Report on Manuscript: “Giant Nonlinear Hall Effect in Isospin Symmetry Broken Bilayer Graphene”

Summary:

The authors report a giant nonlinear Hall conductivity of 9.1 μA V^{-2} in dual-gated Bernal bilayer graphene (BBG), which they attribute to spontaneous isospin symmetry breaking under a perpendicular displacement field. Using quantum oscillation measurements, they identify a partially isospin-polarized (PIP2) state as the origin of this effect. Furthermore, they report an anomalous exponential scaling of the nonlinear conductivity with the linear Drude conductivity at low temperatures. The topic is highly timely, and the experimental observation of a giant nonlinear response in clean BBG is intrinsically interesting and relevant to the community.

However, I have several major concerns that must be addressed before the manuscript can be considered for publication. These concerns primarily stem from the physical implications of valley polarization acting as a time-reversal-symmetry-breaking mechanism.

Response: We thank the reviewer for recognizing the novelty and value of our work. In response to the reviewer’s constructive suggestions and critical questions, we provide point-by-point replies below.

1. Absence of Linear Anomalous Hall Data:
The central interpretive claim of the paper is that the PIP2 state is a valley-polarized state that spontaneously breaks macroscopic time-reversal symmetry (TRS). If TRS is indeed broken, and the Berry curvature flux approaches 2π as claimed in Figure 4b, one would generically expect a finite linear anomalous Hall conductivity (σ_{xy}) in the PIP2 regime. However, the authors neither measure nor discuss the linear Hall conductivity anywhere in the manuscript. To substantiate their claims regarding the symmetry-breaking nature of the PIP2 state, the authors must present the linear Hall

conductivity data in this regime and explicitly discuss its magnitude and behavior in the context of broken TRS.

Response: We thank the reviewer for this important comment. We agree that the linear Hall response is an important quantity when discussing possible macroscopic time-reversal-symmetry breaking in the PIP2 state. In the current version of the manuscript, we did not include the corresponding zero-field linear Hall data, and we will now clarify this point explicitly. In our measurements, we do not resolve a discernible zero-field linear anomalous Hall signal within the experimental sensitivity in the PIP2 regime. The corresponding linear Hall data are shown in Figure R2 in our response to Reviewer 2, Question 2.

We will therefore state this explicitly and discuss it alongside the nonlinear Hall data in the revised manuscript. Nevertheless, the absence of a resolvable linear Hall signal does not by itself rule out a symmetry-broken PIP2 state. A broken-TRS state is a necessary condition for an intrinsic zero-field anomalous Hall response, but not a sufficient condition for a measurable linear Hall conductivity. The observable signal depends on the net first-order Berry-curvature contribution at the actual chemical potential after momentum-space integration, and can remain very small because of partial cancellation between different bands or Fermi-surface sectors. By contrast, the nonlinear Hall effect is a second-order response and can be considerably more sensitive to the detailed Berry-curvature distribution and low-energy asymmetry near the Fermi level.

This distinction is particularly relevant here. In Fig. 4b of our manuscript, the Berry-curvature flux approaching 2π in the PIP2 regime should be understood as a theoretical indicator of strong valley-selective Berry-curvature accumulation associated with the correlated state, rather than as a direct prediction of a large measurable linear anomalous Hall conductivity. In other words, a large Berry-curvature flux and a strong nonlinear Hall response can coexist with an anomalously small linear Hall signal. A similar separation between weak linear Hall response and strong nonlinear Hall response has also been discussed in the recent Bernal bilayer graphene preprint by Chichinadze et al. [arXiv:2411.11156]. We have revised the manuscript accordingly and now explicitly distinguish between the linear Hall response and the nonlinear Hall response when discussing the symmetry-breaking nature of the PIP2 state.

2. Incomplete Treatment of the Nonlinear Conductivity Tensor:
The nonlinear conductivity tensor is a rank-3 tensor. Depending on the exact symmetry of the system, it generally possesses multiple independent components in 2D. The authors utilize a Hall-bar geometry but only measure and report a single transverse component, $\sigma^{2\omega}yxx$. To provide a complete picture of the nonlinear response, the authors should, at a minimum, discuss the expected magnitude of the longitudinal nonlinear response ($\sigma^{2\omega}xxx$) relative to the transverse (Hall) component and explain whether their experimental geometry allows them to extract or bound this value.

Response: We thank the reviewer for this important comment. We agree that the nonlinear conductivity in two dimensions is, in general, a rank-3 tensor and that reporting only a single transverse component does not by itself constitute a complete characterization of the nonlinear response.

In response, we have now examined the longitudinal second-order signal in addition to the transverse one in an additional device.

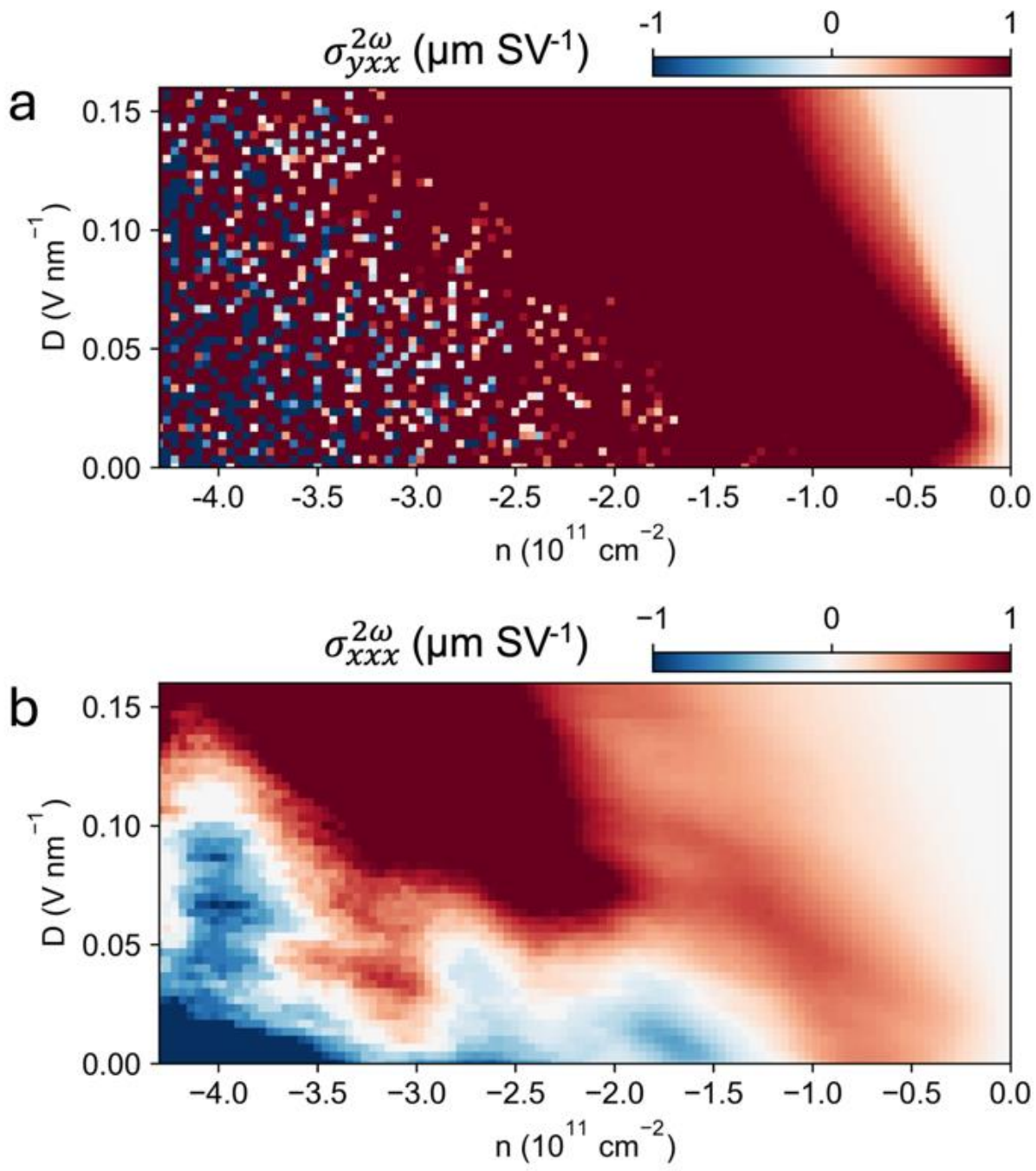

Figure R4. Nonlinear Hall (a) and longitudinal (b) magnitude in (n, D) space of an additional device.

We find that the longitudinal second-order response is clearly finite and broadly distributed over the (n,D) space, whereas the transverse signal is more localized and exhibits stronger phase selectivity. This shows that the nonlinear response in our device is not restricted to a purely Hall-like channel. At the same time, we do not wish to overinterpret the relative magnitude of the longitudinal and transverse signals, because the two channels are not equally clean experimentally.

Qualitatively, the presence of both longitudinal and transverse second-order responses is not unexpected once inversion symmetry is broken. Once inversion symmetry is broken (e.g., by a displacement field), the nonlinear conductivity tensor generally contains multiple independent components. In practice, the transport channel need not be aligned with the crystallographic axes, so both longitudinal and transverse second-order responses can be finite in a Hall-bar measurement. Because of this geometry, both longitudinal and transverse second-order responses can in principle be finite. Microscopically, longitudinal and transverse second-order responses can contain contributions from several distinct intrinsic and extrinsic mechanisms, so the presence of a finite longitudinal channel is not surprising in general. Therefore, the observation that the longitudinal signal is finite while the transverse response is more phase-selective is qualitatively reasonable for nonlinear transport in inversion-broken BBG.

At the same time, we emphasize that the experimental geometry imposes important practical limitations. In a standard Hall-bar configuration, the current is constrained to flow along a single direction, and only a limited subset of tensor combinations can be accessed. Moreover, the longitudinal second-harmonic voltage is significantly more susceptible to parasitic contributions, particularly Joule-heating-induced thermoelectric effects, which generate a 2ω signal with the same symmetry as the intrinsic nonlinear response. As a result, longitudinal second-order signal is generally more difficult to extract cleanly and to interpret quantitatively. In contrast, the transverse channel is less susceptible to such thermal artifacts because of the Hall geometry, making it a more robust and reliable observable in the present device.

We have revised the manuscript to clarify these points explicitly. In particular, we will state that (i) both longitudinal and transverse nonlinear responses are present, (ii) the Hall-bar geometry does not allow a full reconstruction of the rank-3 tensor, and (iii) our analysis focuses on $\sigma_{yxx}^{2\omega}$ because it provides the most phase-sensitive and

experimentally robust signature of the nonlinear response in our device. We hope this expanded discussion provides a more complete and balanced description of the nonlinear response while remaining transparent about the experimental constraints.

3. Incomplete Scaling Analysis and Recent Theoretical Developments:
The authors' interpretation heavily relies on the Berry curvature dipole. I would like to draw the authors' attention to recent theoretical progress on nonlinear responses in materials with broken time-reversal symmetry (see, for example, arXiv:2410.04995). In TRS-broken systems, there are numerous distinct contributions to the nonlinear Hall effect beyond the standard Berry curvature dipole. The authors should perform a more comprehensive scaling-law analysis that accounts for these TRS-breaking-specific contributions. This expanded analysis would significantly improve the theoretical understanding of their anomalous scaling observation and strengthen the manuscript's conclusions.

Response: We thank the reviewer for this insightful comment and for drawing our attention to the recent theoretical developments. We would like to clarify that our manuscript does not rely on the standard Berry-curvature-dipole (BCD) mechanism as a unique microscopic explanation of the observed nonlinear Hall response. Rather, the BCD framework is used in our manuscript as the **conventional semiclassical baseline** against which the experimentally observed scaling is compared. In fact, one of the central results of our work is precisely that the nonlinear response in the PIP2 phase **departs sharply** from this standard picture: while the higher-temperature data follow the expected parabolic trend, the low-temperature response exhibits a clear **exponential enhancement**, which we explicitly identify as a breakdown of the conventional semiclassical scaling relation. We further state in the manuscript that the BCD model is fundamentally limited in the present correlated setting and that our work moves beyond that paradigm. In this sense, we believe the recent theory (arXiv:2410.04995) is consistent with, rather than contradictory to, our present interpretation and we have cited this theory paper in the manuscript.

Recent work has indeed shown that the quadratic nonlinear conductivity generally contains multiple distinct contributions beyond the simplest dipole picture, and that the quantum metric itself is only one part of a much richer second-order response structure. In our manuscript, the physical interpretation of the anomalous

scaling in the PIP_2 regime is already formulated along these broader lines: the low-temperature enhancement is associated with the **TRS-broken valley-polarized state**, in which valley imbalance and the evolution of the Berry-curvature flux enable an additional **skew-scattering-driven** contribution that is absent in the Sym_4 phase. This is why the exponential contribution appears only in PIP_2 and is suppressed once valley degeneracy is restored.

At the same time, we agree with the reviewer that the manuscript would benefit from making this point more explicit. In the revised version, we will clarify that the conventional quadratic scaling is introduced only as a reference for the weakly interacting semiclassical regime, and that our main conclusion is the emergence of an additional TRS-breaking nonlinear channel in the correlated PIP_2 state, rather than a pure BCD interpretation. We will also note that a full microscopic decomposition of all symmetry-allowed second-order contributions in a TRS-broken metal would require substantially more extensive disorder-, angle-, and tensor-resolved measurements than are presently available. Our current goal is therefore more modest and more directly supported by the data: to show experimentally that the nonlinear response in the PIP_2 phase cannot be captured by the standard semiclassical scaling law, and that its behavior is naturally linked to the onset of the interaction-driven valley-polarized state.

With these improvements, I will be in a better position to recommend publication.

Point-by-point responses to reviewers for Manuscript **NCOMMS-26-008629-A**

Reviewer #1 (Remarks to the Author):

The authors have addressed all the concerns and claricactions I raised. Now, I recommend the publication of this work.

Response: We thank the reviewer for positive assessment of our revised manuscript and for recommending publication of our work.

Reviewer #2 (Remarks to the Author):

All previous questions have been addressed satisfactorily. I have only one small remaining concern.

In the Abstract, the concluding sentence states that the effect is "governed by the interplay of quantum geometry and strong electron correlations." The mechanism advanced in this work is skew scattering. While skew scattering can be connected to quantum geometry, the current phrasing is misleading: it suggests that an intrinsic quantum geometric mechanism, such as the Berry curvature dipole or quantum metric, is directly responsible for the observed signal. The authors should revise this sentence and any related passages to explicitly identify skew scattering as the dominant transport mechanism.

With this revision, I recommend publication in Nature Communications.

Response: We thank the reviewer for this constructive suggestion. We agree that the wording should avoid implying that a purely intrinsic quantum-geometric mechanism, such as the Berry curvature dipole or quantum metric, is directly responsible for the observed nonlinear Hall response.

In the revised manuscript, we clarified this point in Abstract by explicitly identifying skew scattering as the dominant transport mechanism. The concluding sentence now reads: “Our findings establish spontaneous isospin symmetry breaking as a novel route to giant nonlinear quantum transport, where interaction-enhanced skew scattering, modulated by the Berry-curvature landscape of the isospin-polarized bands, dominates the observed response.”

Further we also modified related paragraphs throughout the manuscript. Specifically, “This stark deviation signifies a new nonlinear transport regime in which interaction-driven skew scattering no longer follows the conventional semiclassical scaling relations that is dominated by single relaxation time parameter τ” on page 3, “We interpret this anomaly primarily in terms of correlation-enhanced skew scattering associated with the valley-polarized state” on page 6, “In essence, our results reveal how interaction-driven valley polarization can amplify skew scattering through the Berry-curvature landscape of Bernal bilayer graphene” and “In this correlated environment, the Drude conductivity scaling breaks down, and a description incorporating correlation-enhanced skew scattering and the Berry-curvature landscape of the isospin-polarized bands is required” on page 9.

In addition, we also refined the language in the relevant sections of the manuscript to improve clarity and ensure that the proposed mechanism is described more accurately.

We hope these revisions can illustrate the correlation-enhanced skew scattering as the dominant mechanism underlying the observed NLHE.

Reviewer #3 (Remarks to the Author):

The authors clarified almost all the issues. The theoretical explanation a little bit hand waving but can be understood. This is because the non-linear behavior cannot be explained using semi-classical transport argument.

Response: We thank the reviewer for the constructive and positive assessment of our revised manuscript. We agree that a complete microscopic theory of nonlinear transport in the correlation-driven isospin symmetry-broken regime remains challenging, as the observed scaling behavior lies beyond established semiclassical transport descriptions.
